# An essential pathway links FLT3-ITD, HCK and CDK6 in acute myeloid leukemia

**DOI:** 10.18632/oncotarget.9965

**Published:** 2016-06-13

**Authors:** Sophie Lopez, Edwige Voisset, Julie C. Tisserand, Cyndie Mosca, Thomas Prebet, David Santamaria, Patrice Dubreuil, Paulo De Sepulveda

**Affiliations:** ^1^ Inserm, Cancer Research Center of Marseille (CRCM), U1068, Marseille, France; ^2^ Institut Paoli-Calmettes (IPC), Marseille, France; ^3^ Aix-Marseille University, UM 105, Marseille, France; ^4^ CNRS, UMR7258, Marseille, France; ^5^ Present address: Department of Medical and Molecular Genetics, King's College London, Faculty of Life Sciences and Medicine, London, United Kingdom; ^6^ CNIO, Experimental Oncology Group, Madrid, Spain

**Keywords:** AML, protein kinase, oncogene, signaling, palbociclib, SRC

## Abstract

CDK4/CDK6 and RB proteins drive the progression through the G1 phase of the cell cycle. In acute myeloid leukemia (AML), the activity of the CDK/Cyclin D complex is increased. The mechanism involved is unknown, as are the respective roles played by CDK4 or CDK6 in this process. Here, we report that AML cells carrying FLT3-ITD mutations are dependent on CDK6 for cell proliferation while CDK4 is not essential. We showed that FLT3-ITD signaling is responsible for CDK6 overexpression, through a pathway involving the SRC-family kinase HCK. Accordingly, FLT3-ITD failed to transform primary hematopoietic progenitor cells from Cdk6−/− mice. Our results demonstrate that CDK6 is the primary target of CDK4/CDK6 inhibitors in FLT3-ITD positive AML. Furthermore, we delineate an essential protein kinase pathway -FLT3/HCK/CDK6- in the context of AML with FLT3-ITD mutations.

## INTRODUCTION

The FLT3 receptor is mutated in about thirty percent of patients with AML. Most mutations are in-frame internal tandem duplications (ITD) in the juxtamembrane region, which result in constitutive activation of the kinase domain. FLT3-ITD mutations confer a poor prognosis and, in these patients, FLT3 is a validated therapeutic target [1, 2]. Several FLT3 inhibitors have been investigated in clinical trials. However, clinical responses were hampered by the emergence of drug resistance a few months after the initial response [3, 4]. Hence, other drugs in addition to FLT3 inhibitors are critically needed. A better understanding of essential nodes downstream of FLT3-ITD is needed and may lead to alternative therapeutic strategies.

FLT3-ITD signaling results in pleiotropic effects on cell survival, cell proliferation and differentiation. FLT3-ITD activates STAT5, PI3-kinase/AKT and MAP-kinase signaling pathways [5]. Each of these pathways has an impact on the G1/S transition, through control of the cell cycle regulators [6, 7].

The Cyclin D-dependent kinases CDK4 and CDK6 are ubiquitously expressed. They control the G1/S cell cycle progression through phosphorylation of the retinoblastoma (RB) proteins and stabilization of FOXM1 [8, 9]. However, knock-out mouse models have demonstrated that these CDKs are dispensable in most cells [10]. In tumors, CDK4 and CDK6 may be selectively required for oncogenic proliferation as described for CDK4 in breast cancer [11] and melanoma [12], or CDK6 in MLL-rearranged acute myeloid leukemia (AML) [13].

The activity of the CDK4/CDK6/Cyclin D complex is increased in acute myeloid leukemia [14]. In addition, CDK4 was recently proposed as a therapeutic target in AML [15, 16]. However, the molecular mechanisms involved in the activation of the CDK/Cyclin D complex in AML are largely unknown at present time. Thus, it is crucial to determine the oncogenic pathways that regulate this complex.

We describe here a pathway that involves the SRC family kinase HCK and connects FLT3-ITD receptor to the upregulation of CDK6 expression. Furthermore, we demonstrate an essential role for these proteins in leukemogenesis downstream of FLT3-ITD.

## RESULTS

### Screen for essential effectors of oncogenic FLT3-ITD signaling reveals dependence on CDK6

To identify proteins required downstream of the FLT3-ITD oncogenic receptor, we screened a library of siRNAs targeting the entire human kinome (Supplementary Table S1). MV4-11 cells -an AML cell line which endogenously expresses a FLT3-ITD mutation- were used to probe for cell proliferation. Hits that reduced cell proliferation by more than 60% were selected and further investigated. Then, additional controls and filters were applied to narrow the list of candidates towards specific FLT3-ITD effectors (Supplementary Figure S1). To discard false positives, independent siRNAs were tested on MV4-11 cells. To restrict the specificity of inhibition towards the FLT3 oncogene, candidates were then screened using TF-1 cells, an AML cell line that does not harbor FLT3 mutations. Finally, the remaining hits were challenged on another FLT3-ITD AML cell line, MOLM-14.

**Figure 1:**
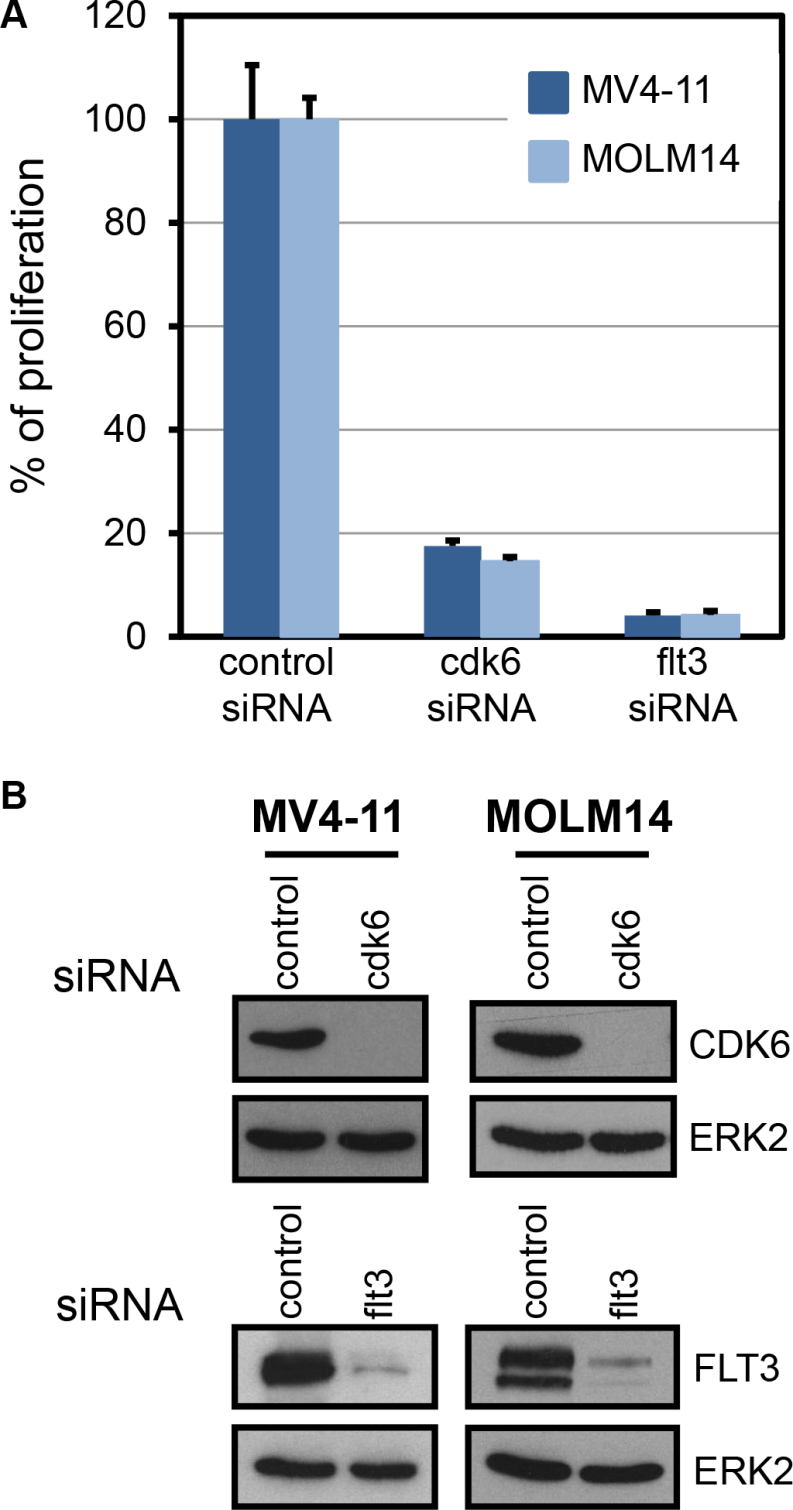
FLT3-ITD cell lines are dependent on CDK6 (**A**) Thymidine incorporation assays were used to evaluate proliferation of MV4-11 and MOLM-14 cells treated with the indicated siRNAs. Histogram shows a representative result (mean ± SD) out of three independent experiments. *P* ≤ 0.001, one way ANOVA multiple comparisons test, between control and cdk6 siRNA. (**B**) Expression of CDK6 and FLT3 proteins in cells treated with cdk6, flt3, or control siRNAs as indicated were controlled by western blot. As a control, expression of ERK2 in the same lysates is shown below each panel.

Only one candidate fulfilled all the criteria described above, the serine/threonine kinase CDK6. Figure 1 shows inhibition of cell proliferation of MV4-11 and MOLM-14 upon downregulation of CDK6 with siRNAs. Importantly, these two cell lines are strictly dependent on FLT3 for cell proliferation (Figure 1A, flt3).

### PD0332991 inhibits proliferation of FLT3-ITD mutated AML cells

**Figure 2:**
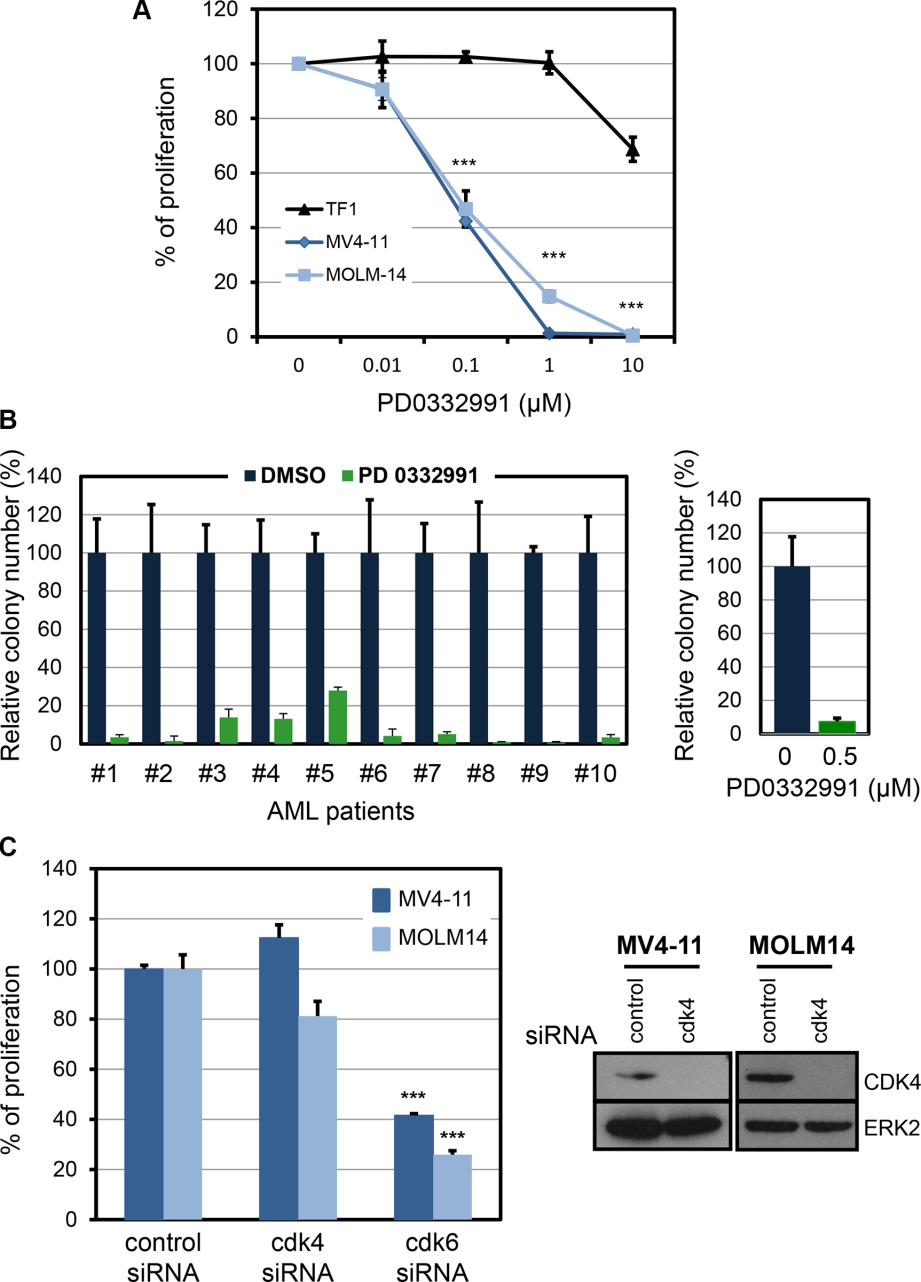
Pharmacological inhibition of CDK6 in human AML cell lines and in primary human FLT3-ITD AML (**A**) Three AML cell lines, TF1, MV4-11 and MOLM-14, were incubated with the selective inhibitor of CDK4/CDK6, PD0332991, at various concentrations for 48 hours. Thymidine incorporation assays were used to evaluate cell proliferation. The graphs are compiled from three independent experiments. ***P ≤ 0.001, one way ANOVA multiple comparisons test. (**B**) Relative number of colonies obtained from primary human FLT3-ITD AML specimens treated without (DMSO) or with 0.5 μM PD0332991. Average effect of PD0332991 on AML samples is shown in the right panel. (**C**) Cell proliferation assay on MV4-11 and MOLM-14 cells treated with the indicated siRNA. The reduced expression of CDK4 protein was controlled by western-blot two days following siRNA transfection. The data in this figure are the mean ± SD. ***P ≤ 0.001, one way ANOVA multiple comparisons test.

To confirm the dependence of MV4-11 and MOLM-14 cells on CDK6, we used PD0332991, a pharmalogical inhibitor of CDK6. Both FLT3 mutated cell lines were inhibited while the non-mutated TF-1 cell line was not (Figure 2A). As a consequence of PD0332991 treatment for 48 hours, MV4-11 cells were blocked in the G1 phase of the cell cycle, with no signs of cell death (Supplementary Figure S2). PD0332991 also inhibited leukemic colony formation from ten AML patients carrying a FLT3-ITD mutation (Figure 2B and Supplementary Table S2).

All CDK6 inhibitors, including PD0332991, also target the closely related kinase CDK4. By using specific siRNAs, we confirmed that CDK4 was dispensable downstream of FLT3-ITD (Figure 2C).

In conclusion, AML cells carrying a FLT3-ITD mutation are dependent on CDK6 but not on the related kinase CDK4 for cell expansion.

### CDK6 is not required downstream of oncogenic KIT receptor

To determine whether CDK6 is a common essential effector of oncogenic tyrosine kinases, we evaluated its contribution downstream of oncogenic KIT, a receptor closely related to FLT3 also involved in hematopoietic neoplasms. CDK6 siRNAs were transfected in HMC1.1, a mast-cell leukemia carrying an endogenous point mutation in the juxtamembrane domain of KIT, and TF-1 cells stably transfected with the classical KIT D816V mutation. While the proliferation of these two cell lines was dependent on KIT, CDK6 expression was dispensable (Supplementary Figure S3). Therefore, we concluded that the dependence to CDK6 is specific to FLT3 oncogenic receptor.

### FLT3-ITD fails to confer cytokine-independence to mouse cdk6 deficient hematopoietic cells

The results described above relied on the use of cell lines that have complex mutation profiles. To address the relationship between CDK6 and FLT3-ITD, independently of other mutations, primary cells were derived from a mouse model deficient for Cdk6. Early hematopoietic progenitors from wild-type (WT) or cdk6−/− mouse bone marrow were isolated and infected with retroviruses encoding FLT3-ITD. Infected cells were then analyzed in colony-forming cell assays.

**Figure 3:**
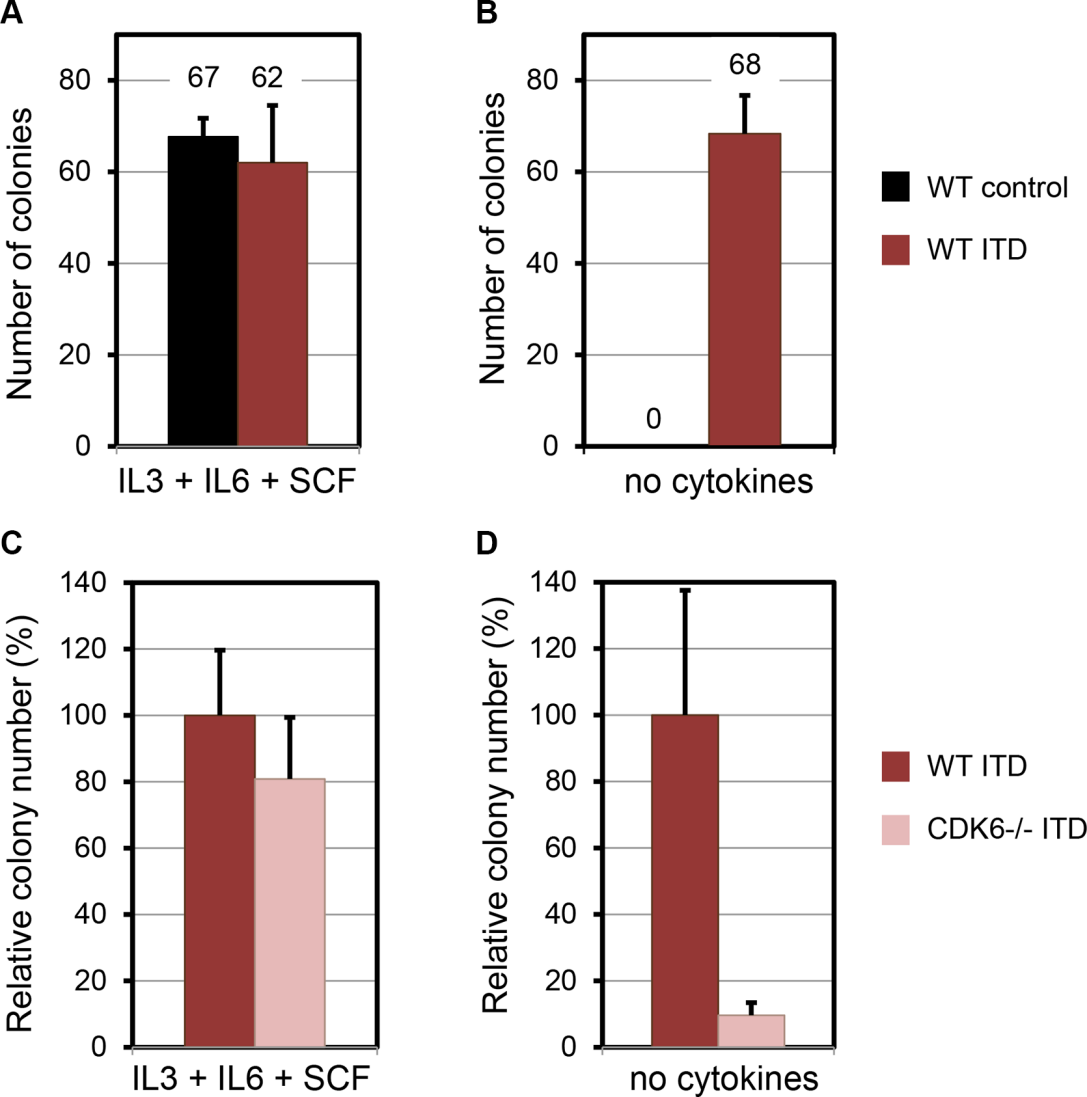
FLT3-ITD requires Cdk6 for transformation of primary murine hematopoietic cells Colony forming cell assay analysis of WT murine bone marrow transduced with control or FLT3-ITD retroviruses, in methylcellulose culture MethoCult^TM^ M3434 (**A**) and M3231 (**B**). (**C** and **D**) Effects of FLT3-ITD on colony formation of Cdk6−/− murine bone marrow cells in methylcellulose culture M3434 (C) and M3231 (D). Data represent the average of three independent experiments (mean ± SD). Statistical analysis using Mann-Whitney: non-significant for (A), and (C); *P* < 0.001 for (B) and (D).

WT bone marrow cells infected either with control or FLT3-ITD vectors gave rise to similar number of colonies in methylcellulose media supplemented with cytokines (Figure 3A). By contrast, only cells infected with FLT3-ITD formed colonies in methycellulose lacking cytokines (Figure 3B). Therefore, the assay using media without cytokines is robust to challenge FLT3-ITD clonogenic potential.

We then compared WT and Cdk6−/− cells infected with FLT3-ITD. Both WT and Cdk6−/− hematopoietic progenitors gave rise to colonies in methylcellulose containing cytokines (Figure 3C). Cdk6−/− progenitors consistently showed slightly less colonies but the difference was not statistically significant. By contrast, the capacity to form colonies from Cdk6−/− cells was greatly impaired in methylcellulose lacking cytokines (100 ± 38 vs 9.1 ± 3.6) (Figure 3D). We concluded that the clonogenic potential conferred by the FLT3-ITD oncoprotein requires Cdk6.

### FLT3-ITD increases CDK6 expression

**Figure 4:**
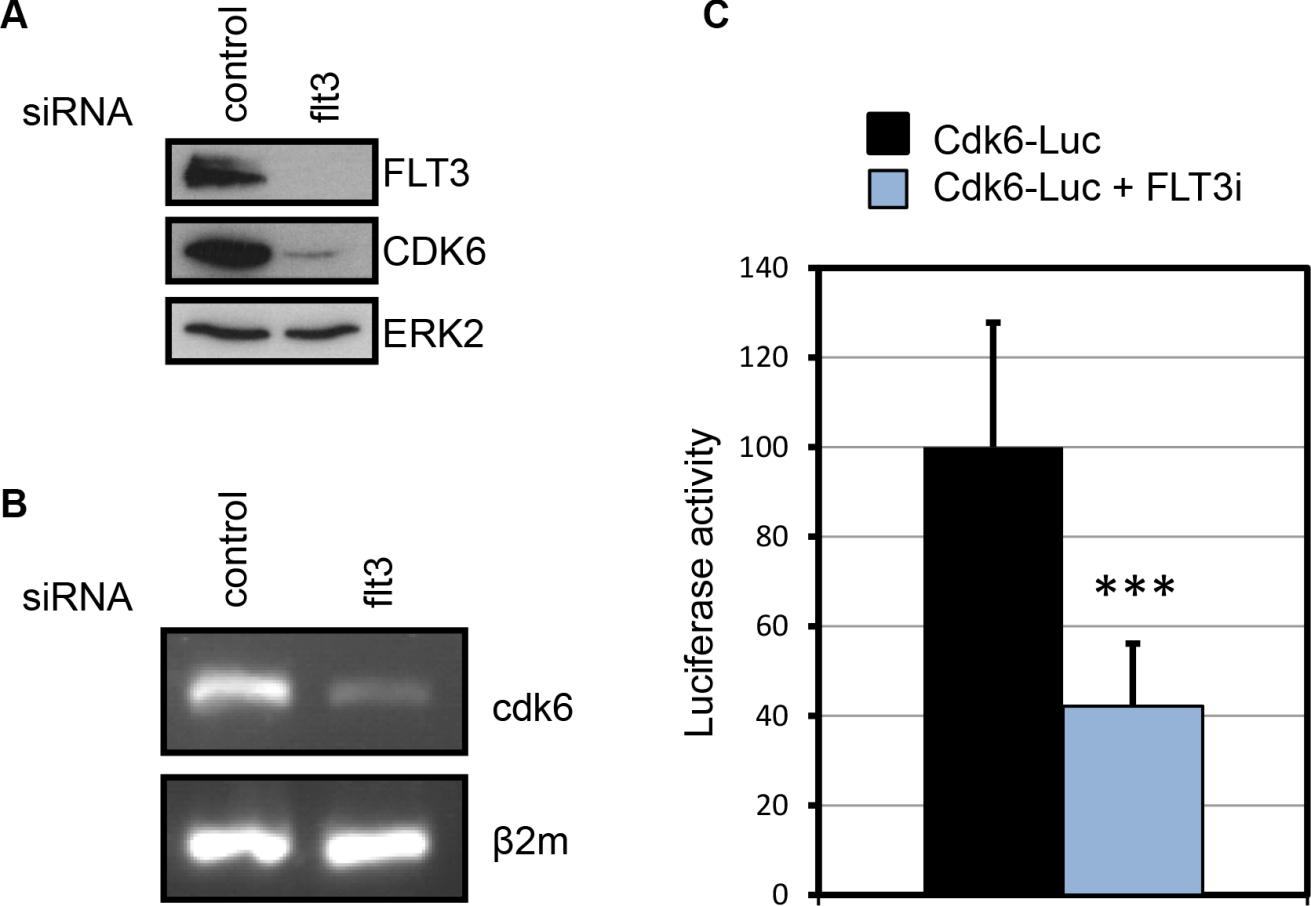
FLT3-ITD increases CDK6 expression (**A**) Expression of CDK6 and FLT3 proteins in MV4-11 cells following FLT3 knockdown by RNA interference. (**B**) Expression of cdk6 mRNA detected by RT-PCR in MV4-11 cells treated with the indicated siRNA. β2 microglobulin was used as a control. (**C**) The activity of a cdk6-luciferase reporter gene was evaluated in MV4-11 cells treated with vehicle or 1 μM of SU11248. Luciferase activity was measured 24 h after electroporation. Data from four independent experiments were compiled (mean ± SD). ***P < 0.0001, Mann-Whitney test.

While performing RNAi experiments, we noticed that knocking down FLT3 reduced the level of CDK6 protein expression (Figure 4A). This suggested that CDK6 expression was in part under the control of FLT3-ITD. This hypothesis was confirmed using an inhibitor of FLT3 catalytic activity (Supplementary Figure S4).

To investigate the mechanism of this regulation, we first analyzed cdk6 transcription since multiple signaling pathways downstream of FLT3-ITD affect gene expression. Cdk6 mRNA level was compared by RT-PCR analysis upon flt3 siRNA treatment. In these experiments, cdk6 mRNA expression was systematically decreased (Figure 4B). Further analyses were performed using a plasmid reporter assay based on the fusion of the cdk6 promoter and the luciferase gene. The reporter plasmid was transfected in MV4-11 cells and luciferase activity was measured twenty-four hours post transfection, in the presence or absence of FLT3 kinase inhibitor. The inhibition of FLT3 resulted in 60% reduction of luciferase activity. Overall, our data indicate that FLT3-ITD increases the level of CDK6 expression at the transcriptional level.

### Identification of the pathway responsible for CDK6 overexpression

**Figure 5:**
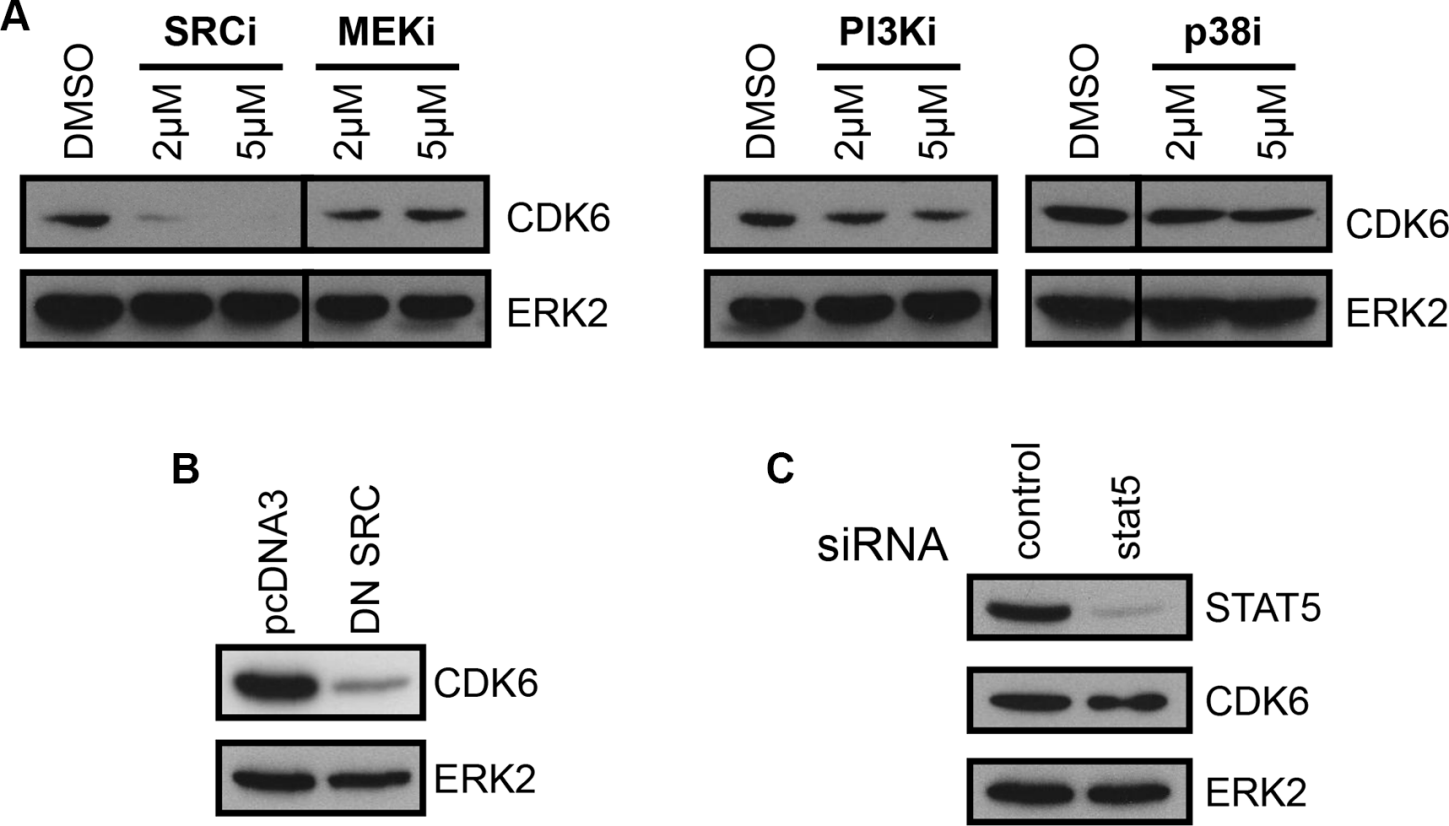
CDK6 expression is dependent on SFKs (**A**) CDK6 protein expression in MV4-11 cells treated for 16 hours with the SRC-family kinase inhibitor SU6656 (SRCi), MEK1/2 inhibitor U0126 (MEKi), PI3-K inhibitor LY294002 (PI3Ki) or p38 inhibitor SB203580 (p38i). The lines in the pictures indicate non-concurrent portions of the same image. (**B**) Expression of CDK6 in MV4-11 cells transfected with a control (pcDNA3) or a dominant-negative SRC (DN SRC) plasmid. (**C**) MV4-11 cells transfected with STAT5 siRNA were analyzed by immunoblotting with anti-CDK6 and anti-STAT5 antibodies. As a control, expression of ERK2 is shown below each panel.

FLT3-ITD activates STAT5, PI3-K, MEK/ERK, p38 and SRC-family kinase pathways. To delineate the signaling network responsible for CDK6 up-regulation, we used pharmacological inhibitors of these pathways. Inhibition of MEK1/2, PI3-K and p38 pathways did not alter expression of CDK6 (Figure 5A). By contrast, inhibition of SRC-family kinases strongly reduced CDK6 expression (Figure 5A, left panel). The use of a SRC dominant-negative mutant confirmed the implication of SRC-family kinases in the regulation of CDK6 expression (Figure 5B). Furthermore, using the hematopoietic colony forming cell assay, we confirmed the requirement for SFKs downstream of FLT3-ITD in the mouse model (Supplementary Figure S5).

STAT5 is a major signal transducer and transcription factor activated downstream of FLT3-ITD receptor. This activation was previously shown to be dependent on SRC-family kinases in murine 32D cells [17, 18] and, we confirmed this observation in the MV4-11 cell model (Supplementary Figure S6). Next, we questioned whether STAT5 was responsible for CDK6 overexpression in this context. RNA interference successfully decreased STAT5 expression, but CDK6 expression remained unchanged (Figure 5C). We concluded that CDK6 expression was controlled by SRC-family kinases, in a STAT5-independent manner.

### HCK is a key effector of FLT3-ITD cell proliferation signaling

In an attempt to decipher which member of the SRC family might be involved downstream of FLT3-ITD, we knocked down the expression of four SRC-family kinases - SRC, FYN, LYN and HCK- that are well expressed in AML.

**Figure 6:**
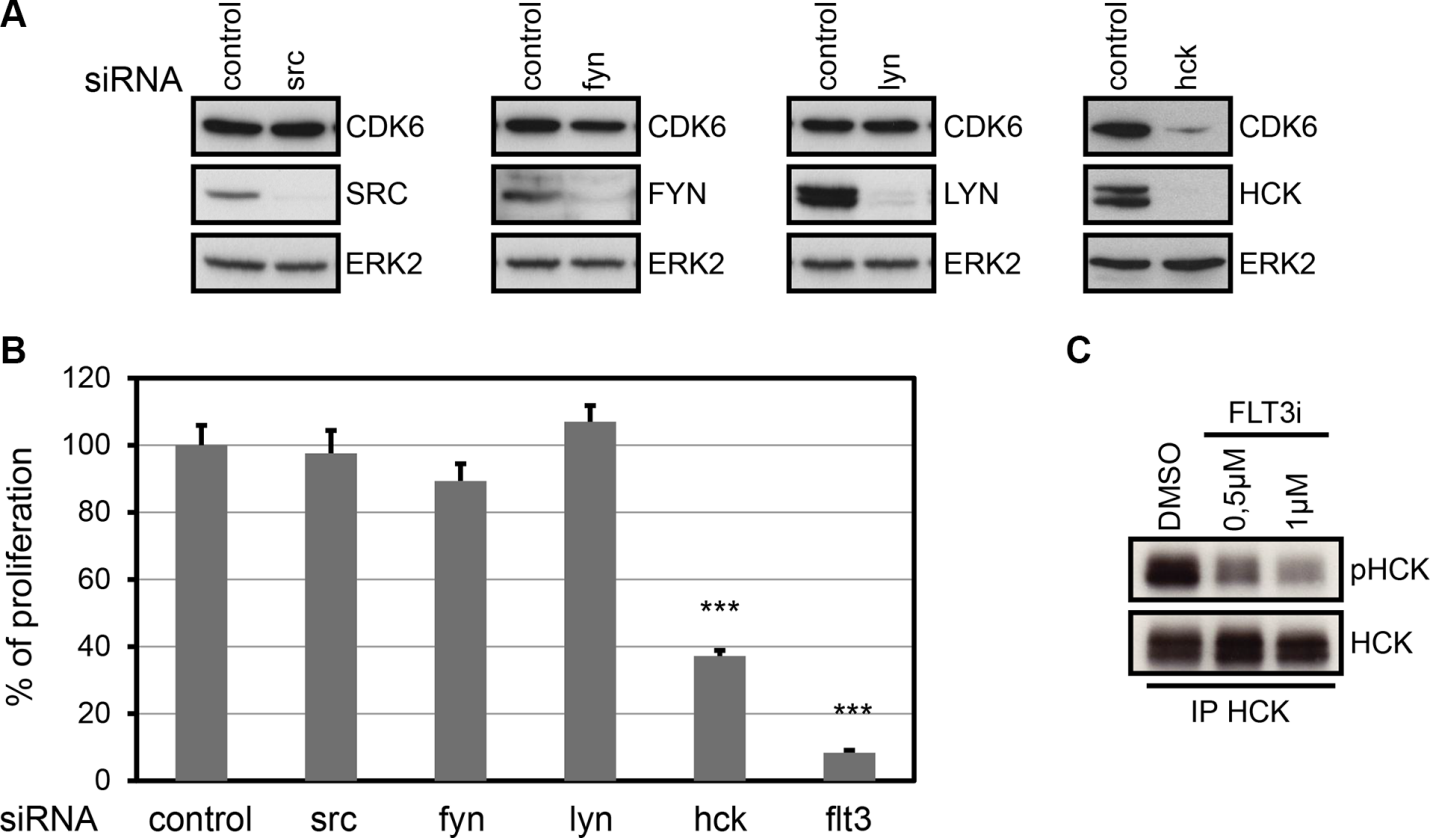
HCK controls CDK6 expression and is required for proliferation of MV4-11 (**A**) CDK6 protein expression in MV4-11 cells following treatment with SRC, FYN, LYN or HCK siRNA. Forty eight hours post electroporation, expression of SRC, FYN, LYN, HCK, CDK6 and ERK2 was evaluated by western-blot. (**B**) MV4-11 cells were treated with the indicated siRNAs. Cell proliferation was quantified using thymidine incorporation assays. Data are the average of three independent experiments (mean ± SD). ***P < 0.001, one way ANOVA multiple comparison test. (**C**) MV4-11 cells were treated with the indicated concentration of SU11248 (FLT3i) for 24 hours. HCK was immunoprecipitated, and an immunoblot was performed with an anti-phospho-Y416-SRC and with anti-HCK antibodies. Representative figures from at least three independent experiments are shown.

RNA interference on SRC, FYN and LYN genes did not modify CDK6 expression (Figure 6A). By contrast, downregulation of HCK resulted in the concomitant decrease of CDK6 expression (Figure 6A, Right panels).

The same RNAi-treated cells were challenged in a cell proliferation assay. As a control, flt3-siRNA strongly impaired cell proliferation, while neither src-, fyn- nor lyn-siRNAs had any effect (Figure 6B). Interestingly, HCK-depleted cells showed 60% reduced proliferation.

Finally, we questioned whether HCK is activated downstream of FLT3-ITD. To address this point, we evaluated the status of HCK major autophosphorylation site in the presence or absence of a FLT3 kinase inhibitor. HCK was indeed phosphorylated on its activation site and this phosphorylation correlated with FLT3 kinase activity (Figure 6C).

In conclusion, these data indicate that HCK is activated downstream of FLT3-ITD, upregulates CDK6 expression and is required for cell proliferation in FLT3-ITD AML cells.

## DISCUSSION

AML is a complex disease with distinct entities/subtypes characterized by molecular mutations and histological criteria. However, irrespective of the mutational status, AML cells show increased activation of the Cyclin D-CDK4/CDK6 complex [14], suggesting that the deregulation of the CDK/RB axis is a hallmark of AML. Cyclin D overexpression, mutations of CDK inhibitors or RB mutations are rare in AML [19, 20]. Our study, together with a recently published study on MLL-rearranged AML [13], suggests that CDK6 plays a central role in this deregulation.

The frequency of CDK4/CDK6 deregulations observed in transformed cells suggests that many cancer cells are dependent on high CDK4/CDK6 activity. By contrast, normal development of most tissues can take place in the absence of Cyclin D-CDK4/CDK6 complexes [10]. Thus, CDK4/6 activity appears as a promising therapeutic target for cancer treatment [20].

MLL translocations in AML induce leukemia through altered gene expression. Recently, MLL-fusion proteins were shown to bind DNA regulatory regions of the CDK6 gene, increase transcription and CDK6 protein expression [13]. Depletion of CDK6 in cell line models, or in AML blasts with MLL oncoproteins, resulted in enhanced cell differentiation and reduced cell proliferation. CDK6 is therefore a promising candidate for therapy in this context.

**Figure 7:**
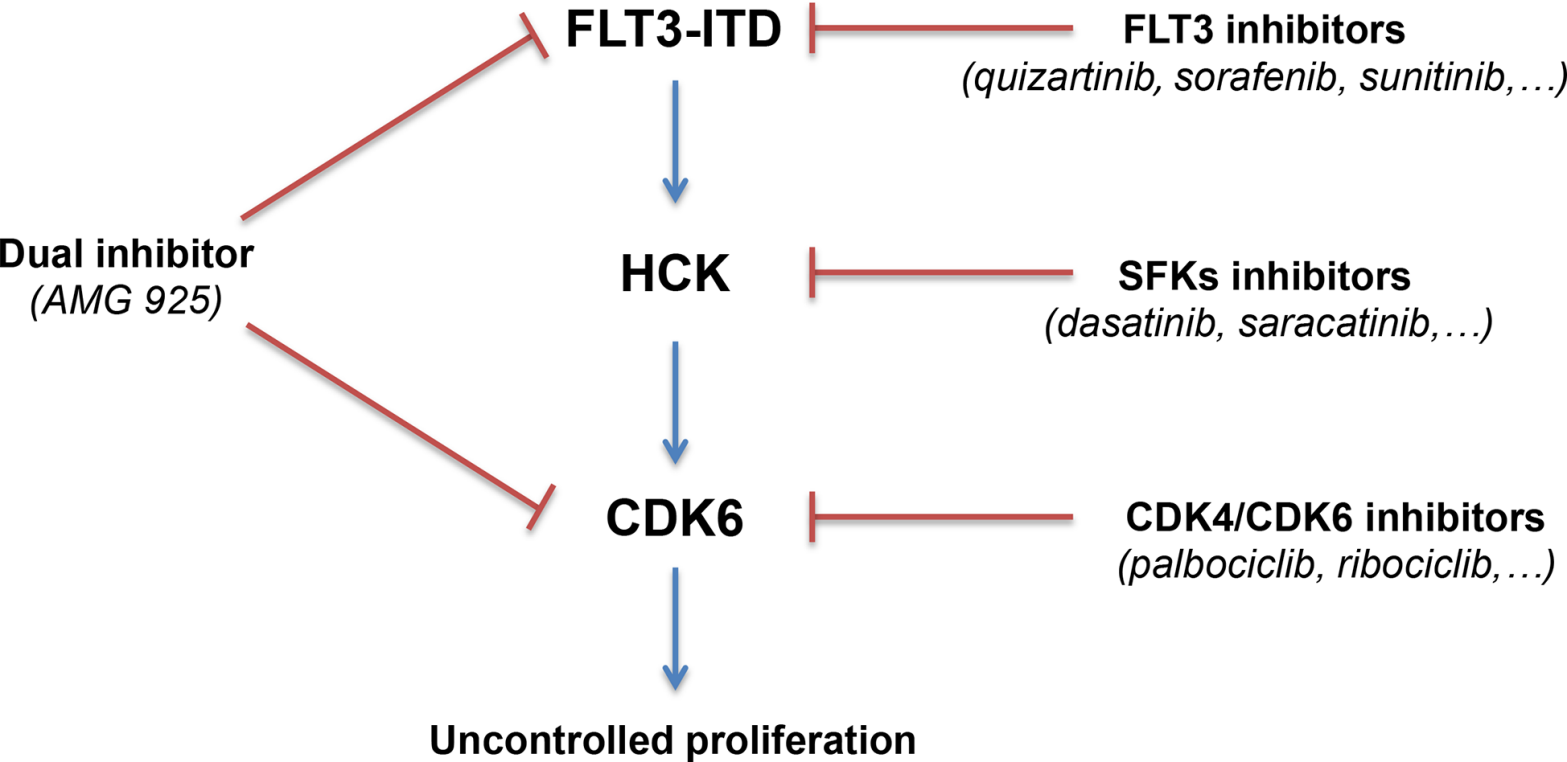
Schematic representation of the FLT3-HCK-CDK6 protein kinase network in FLT3-ITD positive AML Selective drugs available for the clinic are indicated. In addition, AMG 925, a recently described compound with dual-specificity can hit the pathway at two critical points.

AML with FLT3-ITD mutations have poor prognoses. FLT3-ITD catalytic activity can be suppressed using new generation of selective kinase inhibitors. However, the transient initial clinical response is followed by the appearance of resistant blasts. Recent efforts to identify alternative targets in FLT3-ITD AML lead to the identification of candidates such as PU.1 [21], NFATc1 [22], SIRT1 [23], PIM [24], FES [25], SYK [26], and BTK [27]. We describe here an essential pathway downstream of FLT3-ITD in AML cells. This pathway involves two protein kinases, HCK and CDK6, that may be targeted in AML and for which approved selective drugs are available for the clinic (Figure 7). The combination of drugs targeting the FLT3-HCK-CDK6 pathway with classical chemotherapy may represent a rational therapy for clinical trials in FLT3-ITD positive AML.

A novel compound with dual activity against both CDK4/CDK6 and FLT3 has been recently reported [15, 16]. This inhibitor, AMG 925, inhibits AML cell growth in preclinical models and seems to prevent the emergence of resistant clones in FLT3-ITD positive AML cells, a major concern in AML therapy. AMG 925 is referred to as a CDK4/FLT3 dual inhibitor. In the light of our results, we believe that CDK6, and not CDK4, is the target in AML.

Two recent studies reveal a function for CDK6 on murine and human hematopoietic stem cells. Using Cdk6 deficient mice, Scheicher et al. [28] found a role for Cdk6 in stem cell activation. Cdk6 was required for repopulating the hematopoietic lineages in the bone marrow in stress conditions, while steady state hematopoiesis was unaffected. Furthermore, Cdk6 was necessary for propagation of BCR-ABL-induced leukemia by the leukemic stem cells. Another study, comparing short- and long-term repopulating human hematopoietic stem cells reported that CDK6 is involved in quiescence exit [29]. Therefore, besides its function in proliferating AML cells, CDK6 may also act on the compartment of leukemic initiating cells.

In conclusion, our data support the rational for targeting either CDK6 or the pathway responsible for CDK6 activation in FLT3-ITD AML. More broadly, the use of CDK6 inhibitors should be investigated in other AML subtypes. The additional role described for CDK6 in the leukemic stem cell population is of great interest in AML to better understand issues of minimal residual disease, therapy resistance and relapse.

## MATERIALS AND METHODS

### Cell culture

Human AML cell lines MV4-11 and MOLM-14, were provided by Cell Biology Institute, Okayama, Japan. TF-1 KIT^D816V^ cells are TF-1 cells stably transfected with the KIT-D816V receptor [30, 31]. Parental TF-1 cells were grown in the presence of 5 ng/ml of GM-CSF. HMC1.1, a human mast cell line carrying KIT-V560G mutation, was provided by JH Butterfield. All hematopoietic cell lines were maintained in RPMI 1640 medium, supplemented with 10% fetal bovine serum (FBS) (Invitrogen). Phoenix E cells, obtained from ATCC, were grown in DMEM supplemented with 10% FBS and 1 mM sodium pyruvate. All cells were mycoplasma free.

### Cytokines and inhibitors

IL-3, IL-6 and SCF were from Peprotech. Recombinant human GM-CSF was purchased from Berlex. PD0332991/palbociclib was from Selleck Chemicals. SU11248/sunitinib, SU6656, U0126, SB203580, LY294002 were purchased from Calbiochem.

### RNAi screen

The human kinase siRNA library was purchased from Ambion. 10^5^ MV4-11 cells were seeded in a 96-well electroporation plate (Bio-Rad) with 0.03 nmol of siRNA per well. Cells were electroporated at 300 V and 750 μF, and 6,000 cells were replated into duplicate plates in 100 μL of culture media. Cells were incubated for 48 hours at 37°C and pulsed for 6 hours with 0.0185 MBq (0.5 μCi) of [methyl3H]-thymidine (Amersham Biosciences) and thymidine incorporation was measured using a Rackbeta Compact 1212-411 β-counter (LKB).

### siRNA transfection

SiRNAs (0.2 or 0.4 nmol) were mixed with 10^7^ cells in 0.2 (MOLM-14) or 0.5 mL of RPMI 1640 medium in 4 mm gap electroporation cuvettes. Electroporations were carried out at room temperature using a Gene Pulser Electroporator II (Bio-Rad Laboratories) using the following parameters: 300V, 950 μF for MV4-11 cells, 250V, 20 ms for MOLM-14 cells, 250 V, 400 μF for TF-1 cells and 250V, 950 μF for HMC1.1 cells. siRNAs used in the study are listed in Supplementary Table S3.

### Cell proliferation assay

10^4^ cells/well (TF1) or 5 × 10^3^ cells/well (MV4-11 and MOLM-14) were seeded into 96-well plates in 100 μL of culture medium. Cells were incubated for 48 hours at 37°C and pulsed for 6 hours with 0.5 μCi of [methyl3H]-thymidine.

### Immunoprecipitations and Western-blotting

Cell lysates, immunoprecipitations and western-blotting were performed as previously described [32, 33]. Anti-CDK4 (cst-2906), anti-CDK6 (cst-3136), anti-SRC (cst-2110), anti-phospho-STAT5-Y694 (cst-9351), anti-phospho- FLT3-Y591 (cst-3466) and anti-phospho-SRC-Y416 family (cst-2101) were from Cell Signaling Technology. Anti-LYN (sc-7274), anti-FLT3 (sc-480), anti-ERK2 (sc-154), anti-HCK (sc-72), anti-FYN (sc-28791) and anti-STAT5 (sc-835) were from Santa Cruz Biotechnology.

### Mice

WT and cdk6−/− C57Bl/6J mice (from Mariano Barbacid, CNIO, Madrid) were maintained under pathogen-free conditions. Animal experimentations were performed in accordance with protocols approved by the French Ministry of Research and by the ethics committee on animal experimentation C2EA-14.

### Retrovirus production and transduction

The pMIG plasmid containing human FLT3-ITD was previously described [34]. For retrovirus production, 6 μg pMIG-FLT3-ITD or empty vector were transfected into Phoenix E cells using Lipofectamine 2000 (Invitrogen). Supernatants were harvested up to 72 hours after transfection. Bone marrow from 6- to 8-week WT and Cdk6−/− mice was flushed from femurs and tibias, and red blood cells were lysed using ACK Lysing Buffer (Life Technologies). Cells were stained with biotinylated antibodies against CD4, CD8, CD3, CD19, CD11c, DX5, Ter119, CD11b, B220 and Gr1 (BioLegend), incubated with biotin MicroBeads and sorted by Automacs Pro (Miltenyi Biotec). Lineage negative cells were then cultured for 24 hours in Stemspan medium (stem cell technology) with 10 ng/ml murine IL-3, 20 ng/ml murine IL-6, 40 ng/ml murine SCF and 10% FBS. Cells were transduced by two rounds of spin infection at 24 and 48 hours later using retroviral supernatants supplemented with growth factors and 8 μg/mL polybrene (Sigma).

### Colony forming cell assays

3 × 10^4^ cells or 1.5 × 10^5^ cells were mixed with 3 mL of methylcellulose medium (Methocult GF M3434 or MethoCult M3231, StemCell Technologies) and plated into three 35 mm plates. The number of colonies was scored 12 to 14 days later. Human cells from AML patients (3 × 10^5^ cells) were plated in MethoCult H4435 Enriched (StemCell Technologies) with or without PD0332991. Leukemic colony forming units (CFU-L) were scored 5 to 7 days later.

### AML patient samples

AML samples were obtained from the IPC/CRCM Tumour Bank, that operates under authorization # AC-2013-1905 granted by the French Ministry of Research. Patients were appropriately informed and gave writing consent in compliance with French and European regulations. The project was approved by the IPC Institutional Review Board.

### Luciferase promoter-reporter assay

pGL3-CDK6-1.5Kb-Luc, a plasmid containing the *cdk6* promoter, was used for CDK6-luciferase reporter assay [35]. MV4-11 cells were transfected by electroporation and, treated with 1 μM of SU11248 1 hour later. Luciferase activity was measured at 24 hours post electroporation using the Dual-Luciferase reporter assay system (Promega).

### RNA isolation and RT-PCR

Total RNA was isolated using the RNeasy Mini-Kit (Qiagen). For cDNA synthesis, 200 ng of total RNA was used with the Affinity Script multi temperature cDNA synthesis kit (Agilent). Cdk6 primers were 5′-ACTTGAAGAACGGAGGCCGTTT-3′ and 5′-AAGG CCGAAGTCAGCGAGTTTT-3′. *β2*-microglobulin was used as a control (5′-GATGCTGCTTACATGTCTCG-3′ and 5′-CCAGCAGAGAATGGAAAGTC-3′). PCR amplifications were performed on a Mastercycler Nexus gradient (Eppendorf).

## SUPPLEMENTARY MATERIALS FIGURES AND TABLES




