# Supplementary material for: An essential pathway links FLT3-ITD, HCK and CDK6 in acute myeloid leukemia
Source: Oncotarget. 2016 Jun 13;7(32):51163–73. doi: 10.18632/oncotarget.9965 (PMC5239466; doi:10.18632/oncotarget.9965)
Supplement: Supplementary file 1 [file oncotarget-07-51163-s001.pdf]

An essential pathway links FLT3-ITD, HCK and CDK6 in acute myeloid leukemia

Supplementary Materials

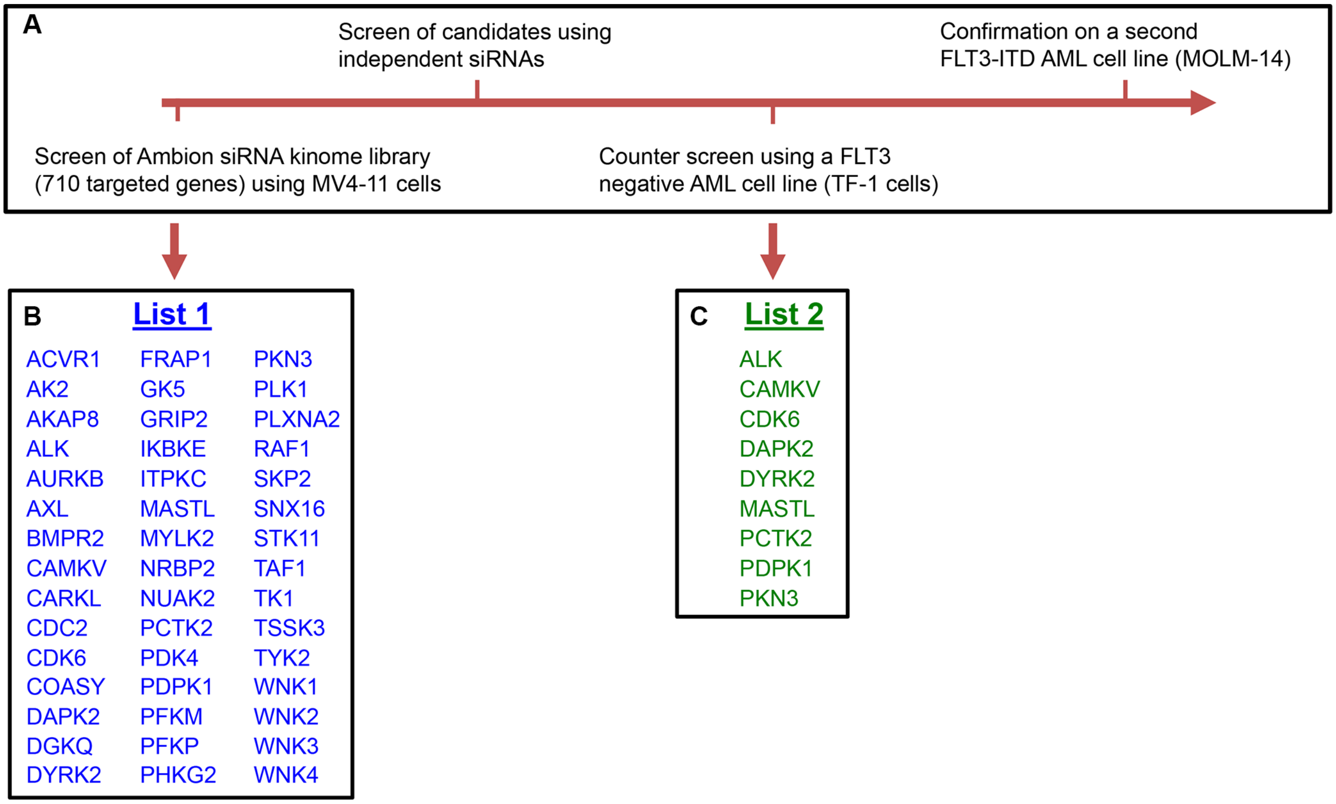

Supplementary Figure S1: Screening strategy. (**A**) Description of the major steps of the screen. (**B**) List of genes that reduced proliferation of MV4-11 cells by at least 60% in the original screen. (**C**) List of genes that were confirmed to inhibit MV4-11 cells but that did not inhibit TF-1 cells.

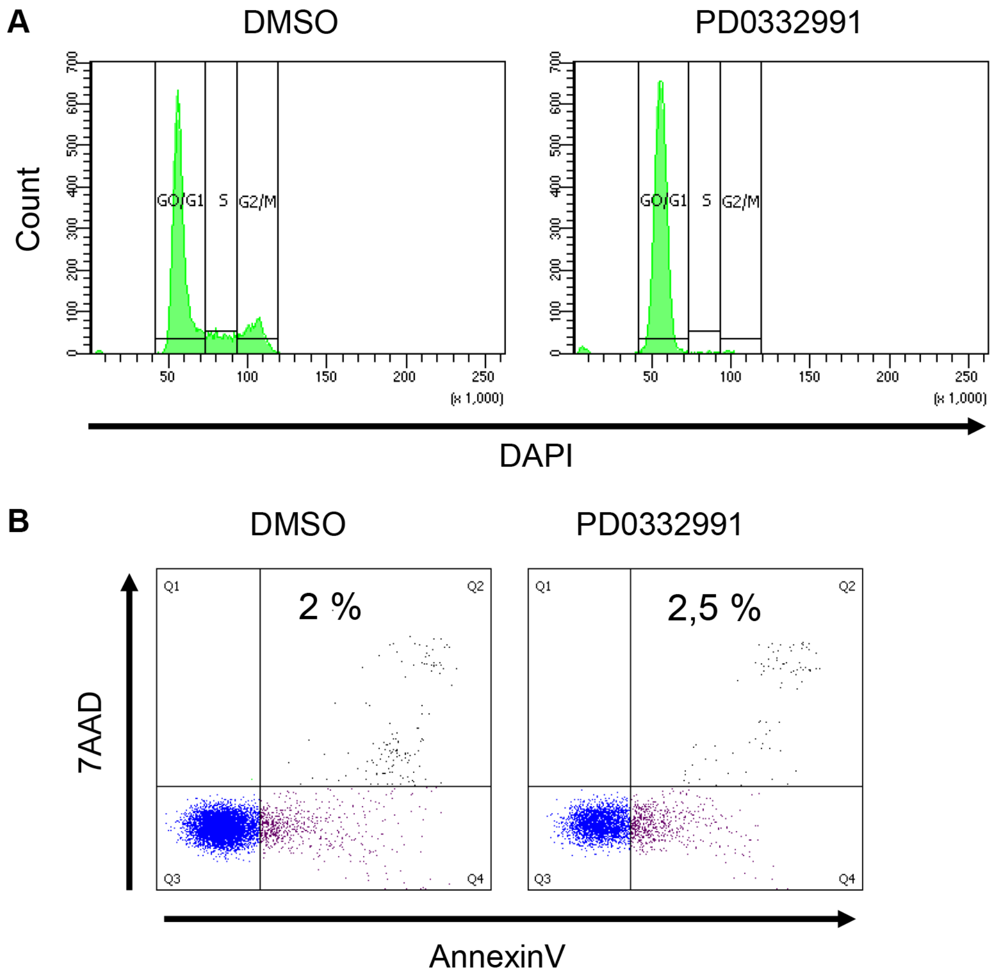

Supplementary Figure S2: Effect of PD0332991 on MV4-11 cell cycle and cell death. (**A**) Cell treated with PD0332991 are blocked in the G1 phase of the cell cycle. Flow cytometry analysis of MV4-11 cells treated with PD0332991 (0,5 µM) for 48 hours and stained with DAPI. (**B**) Analysis of cell death by flow cytometry. MV4-11 cells treated with PD0332991 for 48 hours were stained with 7AAD and Annexin V. The percentage of double positive dead cells is indicated.

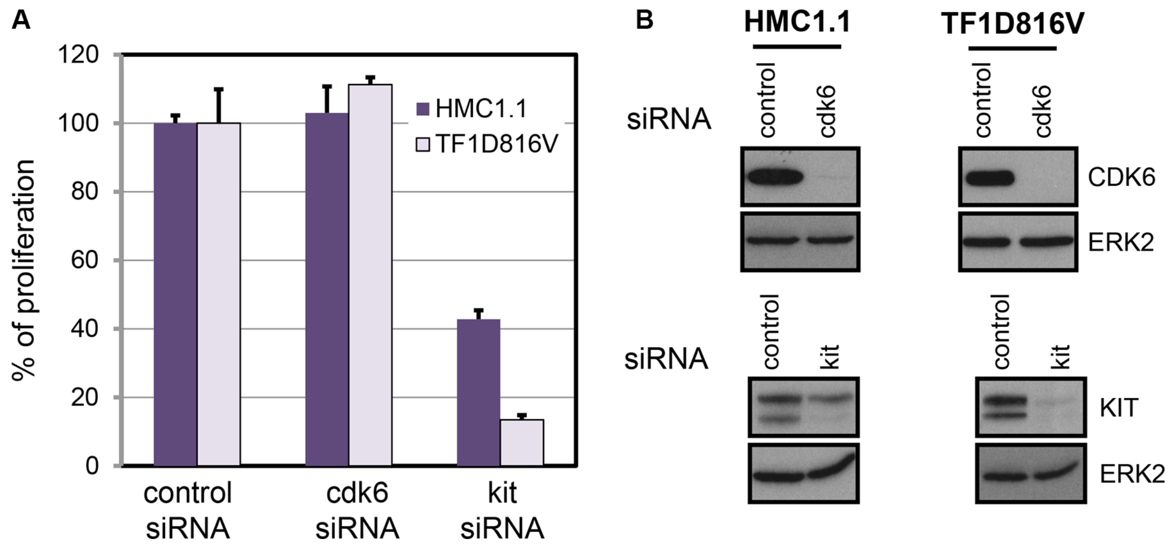

Supplementary Figure S3: KIT mutated cell lines are not dependent on CDK6 expression. (**A**) Thymidine incorporation assays were used to evaluate proliferation of HMC1.1 and TF1D816V cells treated with the indicated siRNAs. The histogram shows a representative result from three independent experiments. (**B**) Expression of CDK6 and KIT proteins in cells treated with control-, cdk6-, or kit- siRNAs as indicated were controlled by western blot. As a control, expression of ERK2 in the same lysates is shown below each panel.

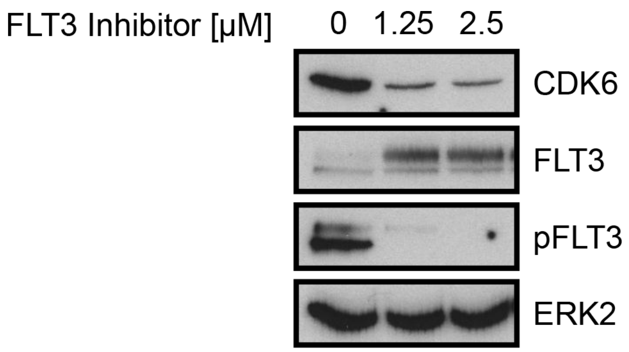

Supplementary Figure S4: CDK6 expression level is dependent on the catalytic activity of FLT3-ITD. CDK6 protein expression was evaluated by western blot in MV4-11 cells treated with SU11248 for 48 hours. Inhibition of FLT3 kinase activity was controlled using a FLT3-Y591 phospho-specific antibody (pFLT3). ERK2 expression is shown as a lysate control.

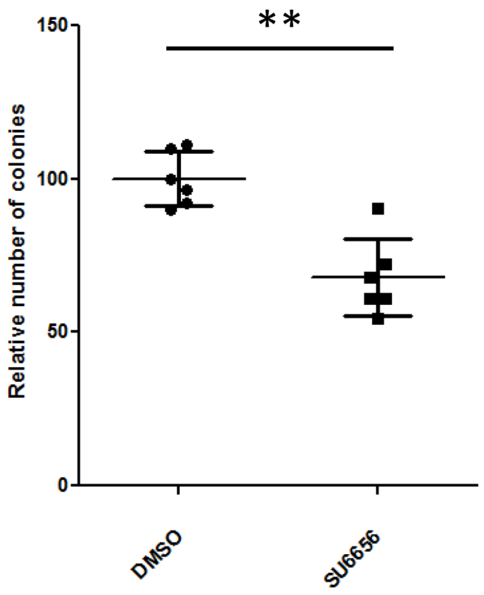

Supplementary Figure S5: FLT3-ITD-mediated colony formation is dependent on SRC-familly kinases. Colony forming assay analysis of wild-type murine bone-marrow transduced with FLT3-ITD retroviruses in the presence of SU6656 (2 µM) or DMSO as a control. Cells were seeded in methylcellulose Methocult M3231 medium. Colonies were counted two weeks later. ***P* ≤ 0,01, Mann-Whitney test.

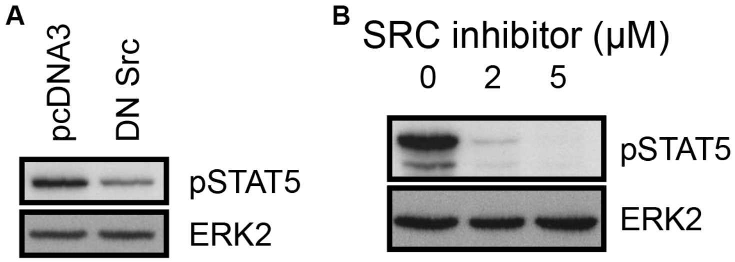

Supplementary Figure S6: STAT5 activation is dependent on SFKs. (**A**) Expression of phosphorylated STAT5 (pSTAT5) in MV4-11 cells transfected with a control (pcDNA3) or a dominant-negative SRC (DN Src) plasmid. (**B**) pSTAT5 protein expression in MV4-11 cells treated with SRC family kinase inhibitor SU6656 or DMSO control (0) for 16 hours. As a loading control, expression of ERK2 in the same membranes is shown below each panel. Representative western blots from three independent experiments are shown.

Supplementary Table S1: List of the 710 genes targeted by the siRNA library. See Supplementary_ Table_S1

Supplementary Table S2: AML samples used in the study[a]

Patient no.	Age	FAB[b]	FLT3	blasts %	Sample origin
1	49	M4	ITD	73	peripheral blood
2	56	M5	ITD	89	peripheral blood
3	47	M5	ITD	91	peripheral blood
4	62	M4	ITD	67	peripheral blood
5	48	M5	ITD	91	peripheral blood
6	20	M4	ITD	67	peripheral blood
7	31	M5	ITD	95	peripheral blood
8	54	M2	ITD	66	peripheral blood
9	58	M4	ITD	82	peripheral blood
10	75	M5	ITD	84	bone marrow

[a]All samples had a normal karyotype.
[b]French American British classification.

Supplementary Table S3: List of siRNAs used in the study

Targeted gene	Species	siRNA sequences (5′ to 3′)	Sources
CDK6	Human	GGCAAAGACCUACUUCUGAAGUGUU<br>GACCACUUACUUGGAUAAAGUUCCA<br>ACCGAGUAGUGCAUCGCGAUCUAAA	Invitrogen[a]
CDK4	Human	GAAAUUGGUGUCGGUGCCUAUGGGA<br>GCACAGUUCGUGAGGUGGCUUUACU<br>UUACCUUGAUCUCCCGGUCAGUUCG	Invitrogen[a]
FYN	Human	GAAGCCCGCUCCUUGACAA<br>GGAGAGACAGGUUACAUUC<br>CGGAUUGGCCCGAUUGAUA<br>GGACUCAUAUGCAAGAUUG	Dharmacon[b]
LYN	Human	GCGACAUGAUUAAACAUUA<br>GUGAUGUUAUUAAGCACUA<br>GAGAUCCAACGUCCAAUAA<br>UUACAUCUCUCCACGAAUC	Dharmacon[b]
SRC	Human	GGGAGAACCUCUAGGCACA<br>GCAGAGAACCCGAGAGGGA<br>CCAAGGGCCUCAACGUGAA<br>GCAGUUGUAUGCUGUGGUU	Dharmacon[b]
HCK	Human	GCCAUCAACUUUGGCUCCUUCACCA<br>AAACUGUCGGUGCCCUGCAUGUCUU<br>GGACAUCAUCGUGGUUGCCCUGUAU	Invitrogen[a]
FLT3	Human	CAAGAAACGACACCGGAUAUU<br>GAAUUUAAGUCGUGUGUUCUU<br>GCAAUGAUAUUUGGGACUAUU<br>CGCAACAGCUUAUGGAAUUUU	Dharmacon[b]
KIT	Human	GGCCGACAAAAGGAGAUCU	Qiagen
Control[c]		UUCUCCGAACGUGUCACGU	Qiagen

[a]Stealth siRNAs.
[b]On-TARGETplus SMART pool siRNAs.
[c]Non targeting siRNA.
