# Supplementary material for: An essential pathway links FLT3-ITD, HCK and CDK6 in acute myeloid leukemia
Source: Oncotarget. 2016 Jun 13;7(32):51163–73. doi: 10.18632/oncotarget.9965 (PMC5239466; doi:10.18632/oncotarget.9965)
Supplement: Supplementary file 2 [file oncotarget-07-51163-s002.docx]

**Supplementary Table S1: List of the 710 genes targeted by the siRNA library**

| Gene symbol | RefSeq accession number | Full gene name | Gene ID |
| --- | --- | --- | --- |
| AAK1 | NM_014911 | AP2 associated kinase 1 | 22848 |
| AATK | NM_001080395 | apoptosis-associated tyrosine kinase | 9625 |
| ABL1 | NM_007313 | v-abl Abelson murine leukemia viral oncogene homolog 1 | 25 |
| ABL2 | NM_007314 | v-abl Abelson murine leukemia viral oncogene homolog 2  (arg, Abelson-related gene) | 27 |
| ACVR1 | NM_001105 | activin A receptor, type I | 90 |
| ACVR1B | NM_020327 | activin A receptor, type IB | 91 |
| ACVR1C | NM_145259 | activin A receptor, type IC | 130399 |
| ACVR2A | NM_001616 | activin A receptor, type IIA | 92 |
| ACVR2B | NM_001106 | activin A receptor, type IIB | 93 |
| ACVRL1 | NM_001077401 | activin A receptor type II-like 1 | 94 |
| ADCK2 | NM_052853 | aarF domain containing kinase 2 | 90956 |
| ADCK4 | NM_024876 | aarF domain containing kinase 4 | 79934 |
| ADCK5 | NM_174922 | aarF domain containing kinase 5 | 203054 |
| ADK | NM_006721 | adenosine kinase | 132 |
| ADPGK | NM_031284 | ADP-dependent glucokinase | 83440 |
| ADRBK1 | NM_001619 | adrenergic, beta, receptor kinase 1 | 156 |
| ADRBK2 | NM_005160 | adrenergic, beta, receptor kinase 2 | 157 |
| AGK | NM_018238 | acylglycerol kinase | 55750 |
| AK1 | NM_000476 | adenylate kinase 1 | 203 |
| AK2 | NM_013411 | adenylate kinase 2 | 204 |
| AK3 | NM_016282 | adenylate kinase 3 | 50808 |
| AK3L1 | NM_001005353 | adenylate kinase 3-like 1 | 205 |
| AK3L2 | NM_001002921 | adenylate kinase 3-like 2 | 387851 |
| AK5 | NM_174858 | adenylate kinase 5 | 26289 |
| AK7 | NM_152327 | adenylate kinase 7 | 122481 |
| AKAP12 | NM_005100 | A kinase (PRKA) anchor protein (gravin) 12 | 9590 |
| AKAP14 | NM_001008534 | A kinase (PRKA) anchor protein 14 | 158798 |
| AKAP7 | NM_016377 | A kinase (PRKA) anchor protein 7 | 9465 |
| AKAP8 | NM_005858 | A kinase (PRKA) anchor protein 8 | 10270 |
| AKAP8L | NM_014371 | A kinase (PRKA) anchor protein 8-like | 26993 |
| AKT1 | NM_001014431 | v-akt murine thymoma viral oncogene homolog 1 | 207 |
| AKT2 | NM_001626 | v-akt murine thymoma viral oncogene homolog 2 | 208 |
| AKT3 | NM_181690 | v-akt murine thymoma viral oncogene homolog 3 (protein kinase B, gamma) | 10000 |
| ALDH18A1 | NM_002860 | aldehyde dehydrogenase 18 family, member A1 | 5832 |
| ALK | NM_004304 | anaplastic lymphoma kinase (Ki-1) | 238 |
| ALPK1 | NM_025144 | alpha-kinase 1 | 80216 |
| ALPK2 | NM_052947 | alpha-kinase 2 | 115701 |
| ALPK3 | NM_020778 | alpha-kinase 3 | 57538 |
| ALS2CR2 | NM_018571 | amyotrophic lateral sclerosis 2 (juvenile) chromosome region, candidate 2 | 55437 |
| ALS2CR7 | NM_139158 | amyotrophic lateral sclerosis 2 (juvenile) chromosome region, candidate 7 | 65061 |
| AMHR2 | NM_020547 | anti-Mullerian hormone receptor, type II | 269 |
| ANKK1 | NM_178510 | ankyrin repeat and kinase domain containing 1 | 255239 |
| ARAF | NM_001654 | v-raf murine sarcoma 3611 viral oncogene homolog | 369 |
| ASB10 | NM_080871 | ankyrin repeat and SOCS box-containing 10 | 136371 |
| ASCIZ | NM_015251 | ATM/ATR-Substrate Chk2-Interacting Zn2+-finger protein | 23300 |
| ATM | NM_138292 | ataxia telangiectasia mutated (includes complementation groups A, C and D) | 472 |
| ATR | NM_001184 | ataxia telangiectasia and Rad3 related | 545 |
| AURKA | NM_003600 | aurora kinase A | 6790 |
| AURKAIP1 | NM_017900 | aurora kinase A interacting protein 1 | 54998 |
| AURKB | NM_004217 | aurora kinase B | 9212 |
| AURKC | NM_003160 | aurora kinase C | 6795 |
| AXL | NM_021913 | AXL receptor tyrosine kinase | 558 |
| BCKDK | NM_005881 | branched chain ketoacid dehydrogenase kinase | 10295 |
| BLK | NM_001715 | B lymphoid tyrosine kinase | 640 |
| BMP2K | NM_198892 | BMP2 inducible kinase | 55589 |
| BMP2KL | XM_936694 | BMP2 inducible kinase-like | 347359 |
| BMPR1A | NM_004329 | bone morphogenetic protein receptor, type IA | 657 |
| BMPR1B | NM_001203 | bone morphogenetic protein receptor, type IB | 658 |
| BMPR2 | NM_001204 | bone morphogenetic protein receptor, type II (serine/threonine kinase) | 659 |
| BMX | NM_001721 | BMX non-receptor tyrosine kinase | 660 |
| BRAF | NM_004333 | v-raf murine sarcoma viral oncogene homolog B1 | 673 |
| BRDG1 | NM_012108 | BCR downstream signaling 1 | 26228 |
| BRSK1 | NM_032430 | BR serine/threonine kinase 1 | 84446 |
| BRSK2 | NM_003957 | BR serine/threonine kinase 2 | 9024 |
| BTK | NM_000061 | Bruton agammaglobulinemia tyrosine kinase | 695 |
| BUB1 | NM_004336 | BUB1 budding uninhibited by benzimidazoles 1 homolog (yeast) | 699 |
| BUB1B | NM_001211 | BUB1 budding uninhibited by benzimidazoles 1 homolog beta (yeast) | 701 |
| C19orf35 | NM_198532 | chromosome 19 open reading frame 35 | 374872 |
| C1orf57 | NM_032324 | chromosome 1 open reading frame 57 | 84284 |
| C21orf7 | NM_020152 | chromosome 21 open reading frame 7 | 56911 |
| C9orf95 | NM_017881 | chromosome 9 open reading frame 95 | 54981 |
| C9orf96 | NM_153710 | chromosome 9 open reading frame 96 | 169436 |
| C9orf98 | NM_152572 | chromosome 9 open reading frame 98 | 158067 |
| CALM1 | NM_006888 | calmodulin 1 (phosphorylase kinase, delta) | 801 |
| CALM2 | NM_001743 | calmodulin 2 (phosphorylase kinase, delta) | 805 |
| CALM3 | NM_005184 | calmodulin 3 (phosphorylase kinase, delta) | 808 |
| CAMK1 | NM_003656 | calcium/calmodulin-dependent protein kinase I | 8536 |
| CAMK1D | NM_020397 | calcium/calmodulin-dependent protein kinase ID | 57118 |
| CAMK1G | NM_020439 | calcium/calmodulin-dependent protein kinase IG | 57172 |
| CAMK2A | NM_015981 | calcium/calmodulin-dependent protein kinase (CaM kinase) II alpha | 815 |
| CAMK2B | NM_001220 | calcium/calmodulin-dependent protein kinase (CaM kinase) II beta | 816 |
| CAMK2D | NM_172115 | calcium/calmodulin-dependent protein kinase (CaM kinase) II delta | 817 |
| CAMK2G | NM_172170 | calcium/calmodulin-dependent protein kinase (CaM kinase) II gamma | 818 |
| CAMK2N1 | NM_018584 | calcium/calmodulin-dependent protein kinase II inhibitor 1 | 55450 |
| CAMK4 | NM_001744 | calcium/calmodulin-dependent protein kinase IV | 814 |
| CAMKK1 | NM_172206 | calcium/calmodulin-dependent protein kinase kinase 1, alpha | 84254 |
| CAMKK2 | NM_172215 | calcium/calmodulin-dependent protein kinase kinase 2, beta | 10645 |
| CAMKV | NM_024046 | CaM kinase-like vesicle-associated | 79012 |
| CARKL | NM_013276 | carbohydrate kinase-like | 23729 |
| CASK | NM_003688 | calcium/calmodulin-dependent serine protein kinase (MAGUK family) | 8573 |
| CCRK | NM_012119 | cell cycle related kinase | 23552 |
| CD2 | NM_001767 | CD2 molecule | 914 |
| CDC2 | NM_001786 | cell division cycle 2, G1 to S and G2 to M | 983 |
| CDC2L1 | NM_033492 | cell division cycle 2-like 1 (PITSLRE proteins) | 984 |
| CDC2L5 | NM_031267 | cell division cycle 2-like 5 (cholinesterase-related cell division controller) | 8621 |
| CDC2L6 | NM_015076 | cell division cycle 2-like 6 (CDK8-like) | 23097 |
| CDC42BPA | NM_003607 | CDC42 binding protein kinase alpha (DMPK-like) | 8476 |
| CDC42BPB | NM_006035 | CDC42 binding protein kinase beta (DMPK-like) | 9578 |
| CDC42BPG | NM_017525 | CDC42 binding protein kinase gamma (DMPK-like) | 55561 |
| CDC42SE2 | NM_001038702 | CDC42 small effector 2 | 56990 |
| CDC7 | NM_003503 | cell division cycle 7 homolog (S. cerevisiae) | 8317 |
| CDK10 | NM_003674 | cyclin-dependent kinase (CDC2-like) 10 | 8558 |
| CDK2 | NM_001798 | cyclin-dependent kinase 2 | 1017 |
| CDK3 | NM_001258 | cyclin-dependent kinase 3 | 1018 |
| CDK4 | NM_000075 | cyclin-dependent kinase 4 | 1019 |
| CDK5 | NM_004935 | cyclin-dependent kinase 5 | 1020 |
| CDK6 | NM_001259 | cyclin-dependent kinase 6 | 1021 |
| CDK7 | NM_001799 | cyclin-dependent kinase 7 (MO15 homolog, Xenopus laevis, cdk-activating kinase) | 1022 |
| CDK8 | NM_001260 | cyclin-dependent kinase 8 | 1024 |
| CDK9 | NM_001261 | cyclin-dependent kinase 9 (CDC2-related kinase) | 1025 |
| CDKL1 | NM_004196 | cyclin-dependent kinase-like 1 (CDC2-related kinase) | 8814 |
| CDKL2 | NM_003948 | cyclin-dependent kinase-like 2 (CDC2-related kinase) | 8999 |
| CDKL3 | NM_016508 | cyclin-dependent kinase-like 3 | 51265 |
| CDKL4 | NM_001009565 | cyclin-dependent kinase-like 4 | 344387 |
| CDKL5 | NM_001037343 | cyclin-dependent kinase-like 5 | 6792 |
| CERK | NM_022766 | ceramide kinase | 64781 |
| CERKL | NM_001030313 | ceramide kinase-like | 375298 |
| CHEK1 | NM_001274 | CHK1 checkpoint homolog (S. pombe) | 1111 |
| CHEK2 | NM_145862 | CHK2 checkpoint homolog (S. pombe) | 11200 |
| CHKA | NM_212469 | choline kinase alpha | 1119 |
| CHUK | NM_001278 | conserved helix-loop-helix ubiquitous kinase | 1147 |
| CIB1 | NM_006384 | calcium and integrin binding 1 (calmyrin) | 10519 |
| CIB4 | NM_001029881 | calcium and integrin binding family member 4 | 130106 |
| CIT | NM_007174 | citron (rho-interacting, serine/threonine kinase 21) | 11113 |
| CKB | NM_001823 | creatine kinase, brain | 1152 |
| CKM | NM_001824 | creatine kinase, muscle | 1158 |
| CKMT1B | NM_020990 | creatine kinase, mitochondrial 1B | 1159 |
| CKMT2 | NM_001825 | creatine kinase, mitochondrial 2 (sarcomeric) | 1160 |
| CLK1 | NM_004071 | CDC-like kinase 1 | 1195 |
| CLK2 | NM_003993 | CDC-like kinase 2 | 1196 |
| CLK3 | NM_001292 | CDC-like kinase 3 | 1198 |
| CLK4 | NM_020666 | CDC-like kinase 4 | 57396 |
| CMPK | NM_016308 | cytidylate kinase | 51727 |
| CNKSR1 | NM_006314 | connector enhancer of kinase suppressor of Ras 1 | 10256 |
| CNKSR3 | NM_173515 | CNKSR family member 3 | 154043 |
| COASY | NM_001042529 | Coenzyme A synthase | 80347 |
| COL4A3BP | NM_031361 | collagen, type IV, alpha 3 (Goodpasture antigen) binding protein | 10087 |
| CRIM1 | NM_016441 | cysteine rich transmembrane BMP regulator 1 (chordin-like) | 51232 |
| CRKRS | NM_016507 | Cdc2-related kinase, arginine/serine-rich | 51755 |
| CSF1R | NM_005211 | colony stimulating factor 1 receptor, formerly McDonough feline sarcoma viral (v-fms) oncogene homolog | 1436 |
| CSK | NM_004383 | c-src tyrosine kinase | 1445 |
| CSNK1A1 | NM_001892 | casein kinase 1, alpha 1 | 1452 |
| CSNK1A1L | NM_145203 | casein kinase 1, alpha 1-like | 122011 |
| CSNK1D | NM_139062 | casein kinase 1, delta | 1453 |
| CSNK1E | NM_001894 | casein kinase 1, epsilon | 1454 |
| CSNK1G1 | NM_022048 | casein kinase 1, gamma 1 | 53944 |
| CSNK1G2 | NM_001319 | casein kinase 1, gamma 2 | 1455 |
| CSNK1G3 | NM_004384 | casein kinase 1, gamma 3 | 1456 |
| CSNK2A1 | NM_001895 | casein kinase 2, alpha 1 polypeptide | 1457 |
| CSNK2A2 | NM_001896 | casein kinase 2, alpha prime polypeptide | 1459 |
| DAK | NM_015533 | dihydroxyacetone kinase 2 homolog (S. cerevisiae) | 26007 |
| DAPK1 | NM_004938 | death-associated protein kinase 1 | 1612 |
| DAPK2 | NM_014326 | death-associated protein kinase 2 | 23604 |
| DAPK3 | NM_001348 | death-associated protein kinase 3 | 1613 |
| DCAKD | NM_024819 | dephospho-CoA kinase domain containing | 79877 |
| DCK | NM_000788 | deoxycytidine kinase | 1633 |
| DCLK1 | NM_004734 | doublecortin-like kinase 1 | 9201 |
| DCLK2 | NM_001040261 | doublecortin-like kinase 2 | 166614 |
| DCLK3 | XM_940612 | doublecortin-like kinase 3 | 85443 |
| DDR1 | NM_001954 | discoidin domain receptor family, member 1 | 780 |
| DDR2 | NM_006182 | discoidin domain receptor family, member 2 | 4921 |
| DGKA | NM_201554 | diacylglycerol kinase, alpha 80kDa | 1606 |
| DGKB | NM_145695 | diacylglycerol kinase, beta 90kDa | 1607 |
| DGKD | NM_003648 | diacylglycerol kinase, delta 130kDa | 8527 |
| DGKE | NM_003647 | diacylglycerol kinase, epsilon 64kDa | 8526 |
| DGKG | NM_001346 | diacylglycerol kinase, gamma 90kDa | 1608 |
| DGKH | NM_152910 | diacylglycerol kinase, eta | 160851 |
| DGKI | NM_004717 | diacylglycerol kinase, iota | 9162 |
| DGKK | NM_001013742 | diacylglycerol kinase, kappa | 139189 |
| DGKQ | NM_001347 | diacylglycerol kinase, theta 110kDa | 1609 |
| DGKZ | NM_201532 | diacylglycerol kinase, zeta 104kDa | 8525 |
| DGUOK | NM_080916 | deoxyguanosine kinase | 1716 |
| DKFZp434B1231 | NM_178275 | eEF1A2 binding protein | 91156 |
| DKFZp761P0423 | XM_937796 | homolog of rat pragma of Rnd2 | 157285 |
| DMPK | NM_001081560 | dystrophia myotonica-protein kinase | 1760 |
| DNAJC6 | NM_014787 | DnaJ (Hsp40) homolog, subfamily C, member 6 | 9829 |
| DOK1 | NM_001381 | docking protein 1, 62kDa (downstream of tyrosine kinase 1) | 1796 |
| DTYMK | NM_012145 | deoxythymidylate kinase (thymidylate kinase) | 1841 |
| DYRK1A | NM_130438 | dual-specificity tyrosine-(Y)-phosphorylation regulated kinase 1A | 1859 |
| DYRK1B | NM_004714 | dual-specificity tyrosine-(Y)-phosphorylation regulated kinase 1B | 9149 |
| DYRK2 | NM_003583 | dual-specificity tyrosine-(Y)-phosphorylation regulated kinase 2 | 8445 |
| DYRK3 | NM_001004023 | dual-specificity tyrosine-(Y)-phosphorylation regulated kinase 3 | 8444 |
| DYRK4 | NM_003845 | dual-specificity tyrosine-(Y)-phosphorylation regulated kinase 4 | 8798 |
| EEF2K | NM_013302 | eukaryotic elongation factor-2 kinase | 29904 |
| EGFR | NM_005228 | epidermal growth factor receptor (erythroblastic leukemia viral (v-erb-b) oncogene homolog, avian) | 1956 |
| EIF2AK1 | NM_014413 | eukaryotic translation initiation factor 2-alpha kinase 1 | 27102 |
| EIF2AK2 | NM_002759 | eukaryotic translation initiation factor 2-alpha kinase 2 | 5610 |
| EIF2AK3 | NM_004836 | eukaryotic translation initiation factor 2-alpha kinase 3 | 9451 |
| EIF2AK4 | NM_001013703 | eukaryotic translation initiation factor 2 alpha kinase 4 | 440275 |
| EPHA1 | NM_005232 | EPH receptor A1 | 2041 |
| EPHA10 | NM_001004338 | EPH receptor A10 | 284656 |
| EPHA2 | NM_004431 | EPH receptor A2 | 1969 |
| EPHA3 | NM_182644 | EPH receptor A3 | 2042 |
| EPHA4 | NM_004438 | EPH receptor A4 | 2043 |
| EPHA5 | NM_004439 | EPH receptor A5 | 2044 |
| EPHA6 | NM_173655 | EPH receptor A6 | 285220 |
| EPHA7 | NM_004440 | EPH receptor A7 | 2045 |
| EPHA8 | NM_001006943 | EPH receptor A8 | 2046 |
| EPHB1 | NM_004441 | EPH receptor B1 | 2047 |
| EPHB2 | NM_017449 | EPH receptor B2 | 2048 |
| EPHB3 | NM_004443 | EPH receptor B3 | 2049 |
| EPHB4 | NM_004444 | EPH receptor B4 | 2050 |
| EPHB6 | NM_004445 | EPH receptor B6 | 2051 |
| ERBB2 | NM_001005862 | v-erb-b2 erythroblastic leukemia viral oncogene homolog 2, neuro/glioblastoma derived oncogene homolog (avian) | 2064 |
| ERBB3 | NM_001005915 | v-erb-b2 erythroblastic leukemia viral oncogene homolog 3 (avian) | 2065 |
| ERBB4 | NM_005235 | v-erb-a erythroblastic leukemia viral oncogene homolog 4 (avian) | 2066 |
| ERN1 | NM_001433 | endoplasmic reticulum to nucleus signalling 1 | 2081 |
| ERN2 | NM_033266 | endoplasmic reticulum to nucleus signalling 2 | 10595 |
| ETNK1 | NM_018638 | ethanolamine kinase 1 | 55500 |
| ETNK2 | NM_018208 | ethanolamine kinase 2 | 55224 |
| FASTK | NM_033015 | Fas-activated serine/threonine kinase | 10922 |
| FER | NM_005246 | fer (fps/fes related) tyrosine kinase (phosphoprotein NCP94) | 2241 |
| FES | NM_002005 | feline sarcoma oncogene | 2242 |
| FGFR1 | NM_023106 | fibroblast growth factor receptor 1 (fms-related tyrosine kinase 2, Pfeiffer syndrome) | 2260 |
| FGFR2 | NM_022970 | fibroblast growth factor receptor 2 | 2263 |
| FGFR3 | NM_000142 | fibroblast growth factor receptor 3 (achondroplasia, thanatophoric dwarfism) | 2261 |
| FGFR4 | NM_002011 | fibroblast growth factor receptor 4 | 2264 |
| FGFRL1 | NM_001004358 | fibroblast growth factor receptor-like 1 | 53834 |
| FGR | NM_001042747 | Gardner-Rasheed feline sarcoma viral (v-fgr) oncogene homolog | 2268 |
| FLJ10986 | NM_018291 | hypothetical protein FLJ10986 | 55277 |
| FLJ25006 | NM_144610 | hypothetical protein FLJ25006 | 124923 |
| FLT1 | NM_002019 | fms-related tyrosine kinase 1 (vascular endothelial growth factor/vascular permeability factor receptor) | 2321 |
| FLT3 | NM_004119 | fms-related tyrosine kinase 3 | 2322 |
| FLT4 | NM_002020 | fms-related tyrosine kinase 4 | 2324 |
| FN3K | NM_022158 | fructosamine 3 kinase | 64122 |
| FN3KRP | NM_024619 | fructosamine-3-kinase-related protein | 79672 |
| FRAP1 | NM_004958 | FK506 binding protein 12-rapamycin associated protein 1 | 2475 |
| FRK | NM_002031 | fyn-related kinase | 2444 |
| FUK | NM_145059 | fucokinase | 197258 |
| FYN | NM_153048 | FYN oncogene related to SRC, FGR, YES | 2534 |
| GAK | XM_001127411 | cyclin G associated kinase | 2580 |
| GALK1 | NM_000154 | galactokinase 1 | 2584 |
| GALK2 | NM_002044 | galactokinase 2 | 2585 |
| GCK | NM_033507 | glucokinase (hexokinase 4, maturity onset diabetes of the young 2) | 2645 |
| GCKR | NM_001486 | glucokinase (hexokinase 4) regulator | 2646 |
| GK | NM_203391 | glycerol kinase | 2710 |
| GK2 | NM_033214 | glycerol kinase 2 | 2712 |
| GK5 | NM_001039547 | glycerol kinase 5 (putative) | 256356 |
| GLYCTK | NM_145262 | glycerate kinase | 132158 |
| GNE | NM_005476 | glucosamine (UDP-N-acetyl)-2-epimerase/N-acetylmannosamine kinase | 10020 |
| GRIP2 | XM_940982 | glutamate receptor interacting protein 2 | 80852 |
| GRK1 | NM_002929 | G protein-coupled receptor kinase 1 | 6011 |
| GRK4 | NM_182982 | G protein-coupled receptor kinase 4 | 2868 |
| GRK5 | NM_005308 | G protein-coupled receptor kinase 5 | 2869 |
| GRK6 | NM_001004106 | G protein-coupled receptor kinase 6 | 2870 |
| GRK7 | NM_139209 | G protein-coupled receptor kinase 7 | 131890 |
| GSG2 | NM_031965 | germ cell associated 2 (haspin) | 83903 |
| GSK3A | NM_019884 | glycogen synthase kinase 3 alpha | 2931 |
| GSK3B | NM_002093 | glycogen synthase kinase 3 beta | 2932 |
| GUK1 | NM_000858 | guanylate kinase 1 | 2987 |
| HCK | NM_002110 | hemopoietic cell kinase | 3055 |
| HGS | NM_004712 | hepatocyte growth factor-regulated tyrosine kinase substrate | 9146 |
| HIPK1 | NM_198269 | homeodomain interacting protein kinase 1 | 204851 |
| HIPK2 | NM_022740 | homeodomain interacting protein kinase 2 | 28996 |
| HIPK3 | NM_001048200 | homeodomain interacting protein kinase 3 | 10114 |
| HIPK4 | NM_144685 | homeodomain interacting protein kinase 4 | 147746 |
| HK1 | NM_033497 | hexokinase 1 | 3098 |
| HK2 | NM_000189 | hexokinase 2 | 3099 |
| HK3 | NM_002115 | hexokinase 3 (white cell) | 3101 |
| HKDC1 | NM_025130 | hexokinase domain containing 1 | 80201 |
| HUNK | NM_014586 | hormonally upregulated Neu-associated kinase | 30811 |
| ICK | NM_014920 | intestinal cell (MAK-like) kinase | 22858 |
| IGF1R | NM_000875 | insulin-like growth factor 1 receptor | 3480 |
| IGSF22 | NM_173588 | immunoglobulin superfamily, member 22 | 283284 |
| IHPK1 | NM_153273 | inositol hexaphosphate kinase 1 | 9807 |
| IHPK2 | NM_001005909 | inositol hexaphosphate kinase 2 | 51447 |
| IHPK3 | NM_054111 | inositol hexaphosphate kinase 3 | 117283 |
| IKBKB | NM_001556 | inhibitor of kappa light polypeptide gene enhancer in B-cells, kinase beta | 3551 |
| IKBKE | NM_014002 | inhibitor of kappa light polypeptide gene enhancer in B-cells, kinase epsilon | 9641 |
| IKBKG | NM_003639 | inhibitor of kappa light polypeptide gene enhancer in B-cells, kinase gamma | 8517 |
| ILK | NM_004517 | integrin-linked kinase | 3611 |
| INSR | NM_000208 | insulin receptor | 3643 |
| INSRR | NM_014215 | insulin receptor-related receptor | 3645 |
| IPMK | NM_152230 | inositol polyphosphate multikinase | 253430 |
| IPPK | NM_022755 | inositol 1,3,4,5,6-pentakisphosphate 2-kinase | 64768 |
| IQCH | NM_001031715 | IQ motif containing H | 64799 |
| IRAK1 | NM_001025242 | interleukin-1 receptor-associated kinase 1 | 3654 |
| IRAK2 | NM_001570 | interleukin-1 receptor-associated kinase 2 | 3656 |
| IRAK3 | NM_007199 | interleukin-1 receptor-associated kinase 3 | 11213 |
| IRAK4 | NM_016123 | interleukin-1 receptor-associated kinase 4 | 51135 |
| ITGB1BP3 | NM_014446 | integrin beta 1 binding protein 3 | 27231 |
| ITK | NM_005546 | IL2-inducible T-cell kinase | 3702 |
| ITPK1 | NM_014216 | inositol 1,3,4-triphosphate 5/6 kinase | 3705 |
| ITPKA | NM_002220 | inositol 1,4,5-trisphosphate 3-kinase A | 3706 |
| ITPKB | NM_002221 | inositol 1,4,5-trisphosphate 3-kinase B | 3707 |
| ITPKC | NM_025194 | inositol 1,4,5-trisphosphate 3-kinase C | 80271 |
| JAK1 | NM_002227 | Janus kinase 1 (a protein tyrosine kinase) | 3716 |
| JAK2 | NM_004972 | Janus kinase 2 (a protein tyrosine kinase) | 3717 |
| JAK3 | NM_000215 | Janus kinase 3 (a protein tyrosine kinase, leukocyte) | 3718 |
| KDR | NM_002253 | kinase insert domain receptor (a type III receptor tyrosine kinase) | 3791 |
| KHK | NM_006488 | ketohexokinase (fructokinase) | 3795 |
| KIAA0999 | NM_025164 | KIAA0999 protein | 23387 |
| KIAA1804 | NM_032435 | mixed lineage kinase 4 | 84451 |
| KIT | NM_000222 | v-kit Hardy-Zuckerman 4 feline sarcoma viral oncogene homolog | 3815 |
| KSR1 | NM_014238 | kinase suppressor of ras 1 | 8844 |
| KSR2 | NM_173598 | kinase suppressor of ras 2 | 283455 |
| LATS1 | NM_004690 | LATS, large tumor suppressor, homolog 1 (Drosophila) | 9113 |
| LATS2 | NM_014572 | LATS, large tumor suppressor, homolog 2 (Drosophila) | 26524 |
| LCK | NM_001042771 | lymphocyte-specific protein tyrosine kinase | 3932 |
| LIMK1 | NM_002314 | LIM domain kinase 1 | 3984 |
| LIMK2 | NM_001031801 | LIM domain kinase 2 | 3985 |
| LMTK2 | NM_014916 | lemur tyrosine kinase 2 | 22853 |
| LMTK3 | XM_936372 | lemur tyrosine kinase 3 | 114783 |
| LOC375133 | NM_199345 | similar to phosphatidylinositol 4-kinase alpha | 375133 |
| LOC390877 | XM_372705 | similar to adenylate kinase 5 isoform 1 | 390877 |
| LRPPRC | NM_133259 | leucine-rich PPR-motif containing | 10128 |
| LRRK1 | NM_024652 | leucine-rich repeat kinase 1 | 79705 |
| LRRK2 | NM_198578 | leucine-rich repeat kinase 2 | 120892 |
| LTK | NM_206961 | leukocyte tyrosine kinase | 4058 |
| LY6G5B | NM_021221 | lymphocyte antigen 6 complex, locus G5B | 58496 |
| LYK5 | NM_001003788 | protein kinase LYK5 | 92335 |
| LYN | NM_002350 | v-yes-1 Yamaguchi sarcoma viral related oncogene homolog | 4067 |
| MADD | NM_130470 | MAP-kinase activating death domain | 8567 |
| MAGI1 | NM_004742 | membrane associated guanylate kinase, WW and PDZ domain containing 1 | 9223 |
| MAGI2 | NM_012301 | membrane associated guanylate kinase, WW and PDZ domain containing 2 | 9863 |
| MAGI3 | NM_152900 | membrane associated guanylate kinase, WW and PDZ domain containing 3 | 260425 |
| MAK | NM_005906 | male germ cell-associated kinase | 4117 |
| MAP2K1 | NM_002755 | mitogen-activated protein kinase kinase 1 | 5604 |
| MAP2K1IP1 | NM_021970 | mitogen-activated protein kinase kinase 1 interacting protein 1 | 8649 |
| MAP2K2 | NM_030662 | mitogen-activated protein kinase kinase 2 | 5605 |
| MAP2K3 | NM_002756 | mitogen-activated protein kinase kinase 3 | 5606 |
| MAP2K4 | NM_003010 | mitogen-activated protein kinase kinase 4 | 6416 |
| MAP2K5 | NM_145160 | mitogen-activated protein kinase kinase 5 | 5607 |
| MAP2K6 | NM_002758 | mitogen-activated protein kinase kinase 6 | 5608 |
| MAP2K7 | NM_145185 | mitogen-activated protein kinase kinase 7 | 5609 |
| MAP3K1 | XM_042066 | mitogen-activated protein kinase kinase kinase 1 | 4214 |
| MAP3K10 | NM_002446 | mitogen-activated protein kinase kinase kinase 10 | 4294 |
| MAP3K11 | NM_002419 | mitogen-activated protein kinase kinase kinase 11 | 4296 |
| MAP3K12 | NM_006301 | mitogen-activated protein kinase kinase kinase 12 | 7786 |
| MAP3K13 | NM_004721 | mitogen-activated protein kinase kinase kinase 13 | 9175 |
| MAP3K14 | NM_003954 | mitogen-activated protein kinase kinase kinase 14 | 9020 |
| MAP3K15 | NM_001001671 | mitogen-activated protein kinase kinase kinase 15 | 389840 |
| MAP3K2 | XM_001128799 | mitogen-activated protein kinase kinase kinase 2 | 10746 |
| MAP3K3 | NM_203351 | mitogen-activated protein kinase kinase kinase 3 | 4215 |
| MAP3K4 | NM_005922 | mitogen-activated protein kinase kinase kinase 4 | 4216 |
| MAP3K5 | NM_005923 | mitogen-activated protein kinase kinase kinase 5 | 4217 |
| MAP3K6 | NM_004672 | mitogen-activated protein kinase kinase kinase 6 | 9064 |
| MAP3K7 | NM_145332 | mitogen-activated protein kinase kinase kinase 7 | 6885 |
| MAP3K7IP2 | NM_015093 | mitogen-activated protein kinase kinase kinase 7 interacting protein 2 | 23118 |
| MAP3K8 | NM_005204 | mitogen-activated protein kinase kinase kinase 8 | 1326 |
| MAP3K9 | NM_033141 | mitogen-activated protein kinase kinase kinase 9 | 4293 |
| MAP4K1 | NM_001042600 | mitogen-activated protein kinase kinase kinase kinase 1 | 11184 |
| MAP4K2 | NM_004579 | mitogen-activated protein kinase kinase kinase kinase 2 | 5871 |
| MAP4K3 | NM_003618 | mitogen-activated protein kinase kinase kinase kinase 3 | 8491 |
| MAP4K4 | NM_145687 | mitogen-activated protein kinase kinase kinase kinase 4 | 9448 |
| MAP4K5 | NM_006575 | mitogen-activated protein kinase kinase kinase kinase 5 | 11183 |
| MAPK1 | NM_138957 | mitogen-activated protein kinase 1 | 5594 |
| MAPK10 | NM_138981 | mitogen-activated protein kinase 10 | 5602 |
| MAPK11 | NM_002751 | mitogen-activated protein kinase 11 | 5600 |
| MAPK12 | NM_002969 | mitogen-activated protein kinase 12 | 6300 |
| MAPK13 | NM_002754 | mitogen-activated protein kinase 13 | 5603 |
| MAPK14 | NM_001315 | mitogen-activated protein kinase 14 | 1432 |
| MAPK15 | NM_139021 | mitogen-activated protein kinase 15 | 225689 |
| MAPK3 | NM_001040056 | mitogen-activated protein kinase 3 | 5595 |
| MAPK4 | NM_002747 | mitogen-activated protein kinase 4 | 5596 |
| MAPK6 | NM_002748 | mitogen-activated protein kinase 6 | 5597 |
| MAPK7 | NM_139034 | mitogen-activated protein kinase 7 | 5598 |
| MAPK8 | NM_002750 | mitogen-activated protein kinase 8 | 5599 |
| MAPK9 | NM_002752 | mitogen-activated protein kinase 9 | 5601 |
| MAPKAPK2 | NM_004759 | mitogen-activated protein kinase-activated protein kinase 2 | 9261 |
| MAPKAPK3 | NM_004635 | mitogen-activated protein kinase-activated protein kinase 3 | 7867 |
| MAPKAPK5 | NM_003668 | mitogen-activated protein kinase-activated protein kinase 5 | 8550 |
| MARK1 | NM_018650 | MAP/microtubule affinity-regulating kinase 1 | 4139 |
| MARK2 | NM_001039468 | MAP/microtubule affinity-regulating kinase 2 | 2011 |
| MARK3 | NM_002376 | MAP/microtubule affinity-regulating kinase 3 | 4140 |
| MARK4 | NM_031417 | MAP/microtubule affinity-regulating kinase 4 | 57787 |
| MAST1 | NM_014975 | microtubule associated serine/threonine kinase 1 | 22983 |
| MAST2 | NM_015112 | microtubule associated serine/threonine kinase 2 | 23139 |
| MAST3 | XM_038150 | microtubule associated serine/threonine kinase 3 | 23031 |
| MASTL | NM_032844 | microtubule associated serine/threonine kinase-like | 84930 |
| MATK | NM_002378 | megakaryocyte-associated tyrosine kinase | 4145 |
| MELK | NM_014791 | maternal embryonic leucine zipper kinase | 9833 |
| MERTK | NM_006343 | c-mer proto-oncogene tyrosine kinase | 10461 |
| MET | NM_000245 | met proto-oncogene (hepatocyte growth factor receptor) | 4233 |
| MFHAS1 | NM_004225 | malignant fibrous histiocytoma amplified sequence 1 | 9258 |
| MGC42105 | NM_153361 | hypothetical protein MGC42105 | 167359 |
| MINK1 | NM_153827 | misshapen-like kinase 1 (zebrafish) | 50488 |
| MKNK1 | NM_003684 | MAP kinase interacting serine/threonine kinase 1 | 8569 |
| MKNK2 | NM_199054 | MAP kinase interacting serine/threonine kinase 2 | 2872 |
| MLCK | NM_182493 | MLCK protein | 91807 |
| MLKL | NM_152649 | mixed lineage kinase domain-like | 197259 |
| MORN1 | NM_024848 | MORN repeat containing 1 | 79906 |
| MOS | NM_005372 | v-mos Moloney murine sarcoma viral oncogene homolog | 4342 |
| MPP1 | NM_002436 | membrane protein, palmitoylated 1, 55kDa | 4354 |
| MPP2 | NM_005374 | membrane protein, palmitoylated 2 (MAGUK p55 subfamily member 2) | 4355 |
| MPP3 | NM_001932 | membrane protein, palmitoylated 3 (MAGUK p55 subfamily member 3) | 4356 |
| MPP4 | NM_033066 | membrane protein, palmitoylated 4 (MAGUK p55 subfamily member 4) | 58538 |
| MPP5 | NM_022474 | membrane protein, palmitoylated 5 (MAGUK p55 subfamily member 5) | 64398 |
| MPP6 | NM_016447 | membrane protein, palmitoylated 6 (MAGUK p55 subfamily member 6) | 51678 |
| MPP7 | NM_173496 | membrane protein, palmitoylated 7 (MAGUK p55 subfamily member 7) | 143098 |
| MRC2 | NM_006039 | mannose receptor, C type 2 | 9902 |
| MST1R | NM_002447 | macrophage stimulating 1 receptor (c-met-related tyrosine kinase) | 4486 |
| MUSK | NM_005592 | muscle, skeletal, receptor tyrosine kinase | 4593 |
| MVK | NM_000431 | mevalonate kinase (mevalonic aciduria) | 4598 |
| MYLK | NM_053028 | myosin, light chain kinase | 4638 |
| MYLK2 | NM_033118 | myosin light chain kinase 2, skeletal muscle | 85366 |
| NADK | NM_023018 | NAD kinase | 65220 |
| NAGK | NM_017567 | N-acetylglucosamine kinase | 55577 |
| NEK1 | NM_012224 | NIMA (never in mitosis gene a)-related kinase 1 | 4750 |
| NEK10 | NM_001031741 | NIMA (never in mitosis gene a)- related kinase 10 | 152110 |
| NEK11 | NM_024800 | NIMA (never in mitosis gene a)- related kinase 11 | 79858 |
| NEK2 | NM_002497 | NIMA (never in mitosis gene a)-related kinase 2 | 4751 |
| NEK3 | NM_002498 | NIMA (never in mitosis gene a)-related kinase 3 | 4752 |
| NEK4 | NM_003157 | NIMA (never in mitosis gene a)-related kinase 4 | 6787 |
| NEK5 | NM_199289 | NIMA (never in mitosis gene a)-related kinase 5 | 341676 |
| NEK6 | NM_014397 | NIMA (never in mitosis gene a)-related kinase 6 | 10783 |
| NEK7 | NM_133494 | NIMA (never in mitosis gene a)-related kinase 7 | 140609 |
| NEK8 | NM_178170 | NIMA (never in mitosis gene a)- related kinase 8 | 284086 |
| NEK9 | NM_033116 | NIMA (never in mitosis gene a)- related kinase 9 | 91754 |
| NLK | NM_016231 | nemo-like kinase | 51701 |
| NME1 | NM_000269 | non-metastatic cells 1, protein (NM23A) expressed in | 4830 |
| NME2 | NM_001018138 | non-metastatic cells 2, protein (NM23B) expressed in | 4831 |
| NME3 | NM_002513 | non-metastatic cells 3, protein expressed in | 4832 |
| NME4 | NM_005009 | non-metastatic cells 4, protein expressed in | 4833 |
| NME5 | NM_003551 | non-metastatic cells 5, protein expressed in (nucleoside-diphosphate kinase) | 8382 |
| NME6 | NM_005793 | non-metastatic cells 6, protein expressed in (nucleoside-diphosphate kinase) | 10201 |
| NME7 | NM_013330 | non-metastatic cells 7, protein expressed in (nucleoside-diphosphate kinase) | 29922 |
| NRBP1 | NM_013392 | nuclear receptor binding protein 1 | 29959 |
| NRBP2 | NM_178564 | nuclear receptor binding protein 2 | 340371 |
| NRGN | NM_006176 | neurogranin (protein kinase C substrate, RC3) | 4900 |
| NRK | NM_198465 | Nik related kinase | 203447 |
| NTRK1 | NM_001007792 | neurotrophic tyrosine kinase, receptor, type 1 | 4914 |
| NTRK2 | NM_001018065 | neurotrophic tyrosine kinase, receptor, type 2 | 4915 |
| NTRK3 | NM_002530 | neurotrophic tyrosine kinase, receptor, type 3 | 4916 |
| NUAK1 | NM_014840 | NUAK family, SNF1-like kinase, 1 | 9891 |
| NUAK2 | NM_030952 | NUAK family, SNF1-like kinase, 2 | 81788 |
| OXSR1 | NM_005109 | oxidative-stress responsive 1 | 9943 |
| PACSIN1 | NM_020804 | protein kinase C and casein kinase substrate in neurons 1 | 29993 |
| PACSIN2 | NM_007229 | protein kinase C and casein kinase substrate in neurons 2 | 11252 |
| PACSIN3 | NM_016223 | protein kinase C and casein kinase substrate in neurons 3 | 29763 |
| PAK1 | NM_002576 | p21/Cdc42/Rac1-activated kinase 1 (STE20 homolog, yeast) | 5058 |
| PAK2 | XM_001126110 | p21 (CDKN1A)-activated kinase 2 | 5062 |
| PAK3 | NM_002578 | p21 (CDKN1A)-activated kinase 3 | 5063 |
| PAK4 | NM_001014834 | p21(CDKN1A)-activated kinase 4 | 10298 |
| PAK6 | NM_020168 | p21(CDKN1A)-activated kinase 6 | 56924 |
| PAK7 | NM_020341 | p21(CDKN1A)-activated kinase 7 | 57144 |
| PANK1 | NM_148977 | pantothenate kinase 1 | 53354 |
| PANK2 | NM_024960 | pantothenate kinase 2 (Hallervorden-Spatz syndrome) | 80025 |
| PANK3 | NM_024594 | pantothenate kinase 3 | 79646 |
| PANK4 | NM_018216 | pantothenate kinase 4 | 55229 |
| PAPSS1 | NM_005443 | 3-phosphoadenosine 5-phosphosulfate synthase 1 | 9061 |
| PAPSS2 | NM_001015880 | 3-phosphoadenosine 5-phosphosulfate synthase 2 | 9060 |
| PASK | NM_015148 | PAS domain containing serine/threonine kinase | 23178 |
| PBK | NM_018492 | PDZ binding kinase | 55872 |
| PCM1 | NM_006197 | pericentriolar material 1 | 5108 |
| PCTK1 | NM_033018 | PCTAIRE protein kinase 1 | 5127 |
| PCTK2 | NM_002595 | PCTAIRE protein kinase 2 | 5128 |
| PCTK3 | NM_002596 | PCTAIRE protein kinase 3 | 5129 |
| PDGFRA | NM_006206 | platelet-derived growth factor receptor, alpha polypeptide | 5156 |
| PDGFRB | NM_002609 | platelet-derived growth factor receptor, beta polypeptide | 5159 |
| PDGFRL | NM_006207 | platelet-derived growth factor receptor-like | 5157 |
| PDIK1L | NM_152835 | PDLIM1 interacting kinase 1 like | 149420 |
| PDK1 | NM_002610 | pyruvate dehydrogenase kinase, isozyme 1 | 5163 |
| PDK2 | NM_002611 | pyruvate dehydrogenase kinase, isozyme 2 | 5164 |
| PDK3 | NM_005391 | pyruvate dehydrogenase kinase, isozyme 3 | 5165 |
| PDK4 | NM_002612 | pyruvate dehydrogenase kinase, isozyme 4 | 5166 |
| PDPK1 | NM_002613 | 3-phosphoinositide dependent protein kinase-1 | 5170 |
| PDXK | NM_003681 | pyridoxal (pyridoxine, vitamin B6) kinase | 8566 |
| PFKL | NM_002626 | phosphofructokinase, liver | 5211 |
| PFKM | NM_000289 | phosphofructokinase, muscle | 5213 |
| PFKP | NM_002627 | phosphofructokinase, platelet | 5214 |
| PFTK1 | NM_012395 | PFTAIRE protein kinase 1 | 5218 |
| PGK1 | NM_000291 | phosphoglycerate kinase 1 | 5230 |
| PGK2 | NM_138733 | phosphoglycerate kinase 2 | 5232 |
| PHKG1 | NM_006213 | phosphorylase kinase, gamma 1 (muscle) | 5260 |
| PHKG2 | NM_000294 | phosphorylase kinase, gamma 2 (testis) | 5261 |
| PI4K2B | NM_018323 | phosphatidylinositol 4-kinase type 2 beta | 55300 |
| PI4KII | NM_018425 | phosphatidylinositol 4-kinase type II | 55361 |
| PICK1 | NM_012407 | protein interacting with PRKCA 1 | 9463 |
| PIK3AP1 | NM_152309 | phosphoinositide-3-kinase adaptor protein 1 | 118788 |
| PIK3C2A | NM_002645 | phosphoinositide-3-kinase, class 2, alpha polypeptide | 5286 |
| PIK3C2B | NM_002646 | phosphoinositide-3-kinase, class 2, beta polypeptide | 5287 |
| PIK3C2G | NM_004570 | phosphoinositide-3-kinase, class 2, gamma polypeptide | 5288 |
| PIK3C3 | NM_002647 | phosphoinositide-3-kinase, class 3 | 5289 |
| PIK3CA | NM_006218 | phosphoinositide-3-kinase, catalytic, alpha polypeptide | 5290 |
| PIK3CB | NM_006219 | phosphoinositide-3-kinase, catalytic, beta polypeptide | 5291 |
| PIK3CD | NM_005026 | phosphoinositide-3-kinase, catalytic, delta polypeptide | 5293 |
| PIK3CG | NM_002649 | phosphoinositide-3-kinase, catalytic, gamma polypeptide | 5294 |
| PIK3R3 | NM_003629 | phosphoinositide-3-kinase, regulatory subunit 3 (p55, gamma) | 8503 |
| PIK3R4 | NM_014602 | phosphoinositide-3-kinase, regulatory subunit 4, p150 | 30849 |
| PIK4CA | NM_002650 | phosphatidylinositol 4-kinase, catalytic, alpha polypeptide | 5297 |
| PIK4CB | NM_002651 | phosphatidylinositol 4-kinase, catalytic, beta polypeptide | 5298 |
| PIM1 | NM_002648 | pim-1 oncogene | 5292 |
| PIM2 | NM_006875 | pim-2 oncogene | 11040 |
| PIM3 | XM_938171 | pim-3 oncogene | 415116 |
| PINK1 | NM_032409 | PTEN induced putative kinase 1 | 65018 |
| PIP5K1A | NM_003557 | phosphatidylinositol-4-phosphate 5-kinase, type I, alpha | 8394 |
| PIP5K1B | NM_003558 | phosphatidylinositol-4-phosphate 5-kinase, type I, beta | 8395 |
| PIP5K1C | NM_012398 | phosphatidylinositol-4-phosphate 5-kinase, type I, gamma | 23396 |
| PIP5K2A | NM_005028 | phosphatidylinositol-4-phosphate 5-kinase, type II, alpha | 5305 |
| PIP5K2B | NM_003559 | phosphatidylinositol-4-phosphate 5-kinase, type II, beta | 8396 |
| PIP5K2C | NM_024779 | phosphatidylinositol-4-phosphate 5-kinase, type II, gamma | 79837 |
| PIP5KL1 | NM_173492 | phosphatidylinositol-4-phosphate 5-kinase-like 1 | 138429 |
| PKLR | NM_181871 | pyruvate kinase, liver and RBC | 5313 |
| PKM2 | NM_182470 | pyruvate kinase, muscle | 5315 |
| PKMYT1 | NM_004203 | protein kinase, membrane associated tyrosine/threonine 1 | 9088 |
| PKN1 | NM_213560 | protein kinase N1 | 5585 |
| PKN2 | NM_006256 | protein kinase N2 | 5586 |
| PKN3 | NM_013355 | protein kinase N3 | 29941 |
| PLK1 | NM_005030 | polo-like kinase 1 (Drosophila) | 5347 |
| PLK2 | NM_006622 | polo-like kinase 2 (Drosophila) | 10769 |
| PLK3 | NM_004073 | polo-like kinase 3 (Drosophila) | 1263 |
| PLK4 | NM_014264 | polo-like kinase 4 (Drosophila) | 10733 |
| PLXNA1 | NM_032242 | plexin A1 | 5361 |
| PLXNA2 | NM_025179 | plexin A2 | 5362 |
| PLXNA3 | NM_017514 | plexin A3 | 55558 |
| PLXNA4B | NM_181775 | plexin A4, B | 91584 |
| PLXNB1 | NM_002673 | plexin B1 | 5364 |
| PLXNB2 | XM_371474 | plexin B2 | 23654 |
| PLXNB3 | NM_005393 | plexin B3 | 5365 |
| PLXNC1 | NM_005761 | plexin C1 | 10154 |
| PLXND1 | NM_015103 | plexin D1 | 23129 |
| PMVK | NM_006556 | phosphomevalonate kinase | 10654 |
| PNCK | NM_001039582 | pregnancy upregulated non-ubiquitously expressed CaM kinase | 139728 |
| PNKP | NM_007254 | polynucleotide kinase 3-phosphatase | 11284 |
| PRKAA1 | NM_006251 | protein kinase, AMP-activated, alpha 1 catalytic subunit | 5562 |
| PRKAA2 | NM_006252 | protein kinase, AMP-activated, alpha 2 catalytic subunit | 5563 |
| PRKACA | NM_002730 | protein kinase, cAMP-dependent, catalytic, alpha | 5566 |
| PRKACB | NM_207578 | protein kinase, cAMP-dependent, catalytic, beta | 5567 |
| PRKACG | NM_002732 | protein kinase, cAMP-dependent, catalytic, gamma | 5568 |
| PRKCA | NM_002737 | protein kinase C, alpha | 5578 |
| PRKCB1 | NM_212535 | protein kinase C, beta 1 | 5579 |
| PRKCD | NM_212539 | protein kinase C, delta | 5580 |
| PRKCDBP | NM_145040 | protein kinase C, delta binding protein | 112464 |
| PRKCE | NM_005400 | protein kinase C, epsilon | 5581 |
| PRKCG | NM_002739 | protein kinase C, gamma | 5582 |
| PRKCH | NM_006255 | protein kinase C, eta | 5583 |
| PRKCI | NM_002740 | protein kinase C, iota | 5584 |
| PRKCQ | NM_006257 | protein kinase C, theta | 5588 |
| PRKCSH | NM_002743 | protein kinase C substrate 80K-H | 5589 |
| PRKCZ | NM_001033582 | protein kinase C, zeta | 5590 |
| PRKD1 | NM_002742 | protein kinase D1 | 5587 |
| PRKD2 | NM_001079880 | protein kinase D2 | 25865 |
| PRKD3 | NM_005813 | protein kinase D3 | 23683 |
| PRKDC | NM_001081640 | protein kinase, DNA-activated, catalytic polypeptide | 5591 |
| PRKG1 | NM_006258 | protein kinase, cGMP-dependent, type I | 5592 |
| PRKG2 | NM_006259 | protein kinase, cGMP-dependent, type II | 5593 |
| PRKX | NM_005044 | protein kinase, X-linked | 5613 |
| PRKY | NM_002760 | protein kinase, Y-linked | 5616 |
| PRPF4B | NM_003913 | PRP4 pre-mRNA processing factor 4 homolog B (yeast) | 8899 |
| PRPS1 | NM_002764 | phosphoribosyl pyrophosphate synthetase 1 | 5631 |
| PRPS1L1 | NM_175886 | phosphoribosyl pyrophosphate synthetase 1-like 1 | 221823 |
| PRPS2 | NM_001039091 | phosphoribosyl pyrophosphate synthetase 2 | 5634 |
| PRPSAP1 | NM_002766 | phosphoribosyl pyrophosphate synthetase-associated protein 1 | 5635 |
| PRPSAP2 | NM_002767 | phosphoribosyl pyrophosphate synthetase-associated protein 2 | 5636 |
| PSKH1 | NM_006742 | protein serine kinase H1 | 5681 |
| PSKH2 | NM_033126 | protein serine kinase H2 | 85481 |
| PTCD2 | NM_024754 | pentatricopeptide repeat domain 2 | 79810 |
| PTK2 | NM_153831 | PTK2 protein tyrosine kinase 2 | 5747 |
| PTK2B | NM_173176 | PTK2B protein tyrosine kinase 2 beta | 2185 |
| PTK6 | NM_005975 | PTK6 protein tyrosine kinase 6 | 5753 |
| PTK7 | NM_002821 | PTK7 protein tyrosine kinase 7 | 5754 |
| PXK | NM_017771 | PX domain containing serine/threonine kinase | 54899 |
| RAF1 | NM_002880 | v-raf-1 murine leukemia viral oncogene homolog 1 | 5894 |
| RAGE | NM_014226 | renal tumor antigen | 5891 |
| RBKS | NM_022128 | ribokinase | 64080 |
| RET | NM_020630 | ret proto-oncogene | 5979 |
| RFK | NM_018339 | riboflavin kinase | 55312 |
| RIOK1 | NM_031480 | RIO kinase 1 (yeast) | 83732 |
| RIOK2 | NM_018343 | RIO kinase 2 (yeast) | 55781 |
| RIOK3 | NM_003831 | RIO kinase 3 (yeast) | 8780 |
| RIPK1 | NM_003804 | receptor (TNFRSF)-interacting serine-threonine kinase 1 | 8737 |
| RIPK2 | NM_003821 | receptor-interacting serine-threonine kinase 2 | 8767 |
| RIPK3 | NM_006871 | receptor-interacting serine-threonine kinase 3 | 11035 |
| RIPK4 | NM_020639 | receptor-interacting serine-threonine kinase 4 | 54101 |
| RIPK5 | NM_015375 | receptor interacting protein kinase 5 | 25778 |
| RNASEL | NM_021133 | ribonuclease L (2,5-oligoisoadenylate synthetase-dependent) | 6041 |
| ROCK1 | NM_005406 | Rho-associated, coiled-coil containing protein kinase 1 | 6093 |
| ROCK2 | NM_004850 | Rho-associated, coiled-coil containing protein kinase 2 | 9475 |
| ROR1 | NM_001083592 | receptor tyrosine kinase-like orphan receptor 1 | 4919 |
| ROR2 | NM_004560 | receptor tyrosine kinase-like orphan receptor 2 | 4920 |
| ROS1 | NM_002944 | v-ros UR2 sarcoma virus oncogene homolog 1 (avian) | 6098 |
| RP11-145H9.1 | NM_001012418 | hypothetical protein LOC340156 | 340156 |
| RP2 | NM_006915 | retinitis pigmentosa 2 (X-linked recessive) | 6102 |
| RP6-213H19.1 | NM_001042453 | serine/threonine protein kinase MST4 | 51765 |
| RPS6KA1 | NM_001006665 | ribosomal protein S6 kinase, 90kDa, polypeptide 1 | 6195 |
| RPS6KA2 | NM_001006932 | ribosomal protein S6 kinase, 90kDa, polypeptide 2 | 6196 |
| RPS6KA3 | NM_004586 | ribosomal protein S6 kinase, 90kDa, polypeptide 3 | 6197 |
| RPS6KA4 | NM_003942 | ribosomal protein S6 kinase, 90kDa, polypeptide 4 | 8986 |
| RPS6KA5 | NM_182398 | ribosomal protein S6 kinase, 90kDa, polypeptide 5 | 9252 |
| RPS6KA6 | NM_014496 | ribosomal protein S6 kinase, 90kDa, polypeptide 6 | 27330 |
| RPS6KB1 | NM_003161 | ribosomal protein S6 kinase, 70kDa, polypeptide 1 | 6198 |
| RPS6KB2 | NM_003952 | ribosomal protein S6 kinase, 70kDa, polypeptide 2 | 6199 |
| RPS6KC1 | NM_012424 | ribosomal protein S6 kinase, 52kDa, polypeptide 1 | 26750 |
| RPS6KL1 | NM_031464 | ribosomal protein S6 kinase-like 1 | 83694 |
| RYK | NM_001005861 | RYK receptor-like tyrosine kinase | 6259 |
| SBK1 | XM_937568 | SH3-binding domain kinase 1 | 388228 |
| SCGB2A1 | NM_002407 | secretoglobin, family 2A, member 1 | 4246 |
| SCYL1 | NM_001048218 | SCY1-like 1 (S. cerevisiae) | 57410 |
| SCYL2 | NM_017988 | SCY1-like 2 (S. cerevisiae) | 55681 |
| SCYL3 | NM_181093 | SCY1-like 3 (S. cerevisiae) | 57147 |
| SGK | NM_005627 | serum/glucocorticoid regulated kinase | 6446 |
| SGK2 | NM_170693 | serum/glucocorticoid regulated kinase 2 | 10110 |
| SGK269 | XM_370878 | NKF3 kinase family member | 79834 |
| SGK3 | NM_001033578 | serum/glucocorticoid regulated kinase family, member 3 | 23678 |
| SH3BP4 | NM_014521 | SH3-domain binding protein 4 | 23677 |
| SH3BP5 | NM_001018009 | SH3-domain binding protein 5 (BTK-associated) | 9467 |
| SH3BP5L | NM_030645 | SH3-binding domain protein 5-like | 80851 |
| SKAP1 | NM_001075099 | src kinase associated phosphoprotein 1 | 8631 |
| SKP2 | NM_032637 | S-phase kinase-associated protein 2 (p45) | 6502 |
| SLAMF6 | NM_052931 | SLAM family member 6 | 114836 |
| SLK | NM_014720 | STE20-like kinase (yeast) | 9748 |
| SMG1 | NM_015092 | PI-3-kinase-related kinase SMG-1 | 23049 |
| SNF1LK | NM_173354 | SNF1-like kinase | 150094 |
| SNF1LK2 | NM_015191 | SNF1-like kinase 2 | 23235 |
| SNRK | NM_017719 | SNF related kinase | 54861 |
| SNX16 | NM_022133 | sorting nexin 16 | 64089 |
| SPHK1 | NM_182965 | sphingosine kinase 1 | 8877 |
| SPHK2 | NM_020126 | sphingosine kinase 2 | 56848 |
| SRC | NM_198291 | v-src sarcoma (Schmidt-Ruppin A-2) viral oncogene homolog (avian) | 6714 |
| SRMS | NM_080823 | src-related kinase lacking C-terminal regulatory tyrosine and N-terminal myristylation sites | 6725 |
| SRPK1 | NM_003137 | SFRS protein kinase 1 | 6732 |
| SRPK2 | NM_182691 | SFRS protein kinase 2 | 6733 |
| SRPK3 | NM_014370 | SFRS protein kinase 3 | 26576 |
| STAP2 | NM_017720 | signal-transducing adaptor protein-2 | 55620 |
| STK10 | NM_005990 | serine/threonine kinase 10 | 6793 |
| STK11 | NM_000455 | serine/threonine kinase 11 | 6794 |
| STK11IP | NM_052902 | serine/threonine kinase 11 interacting protein | 114790 |
| STK16 | NM_001008910 | serine/threonine kinase 16 | 8576 |
| STK17A | NM_004760 | serine/threonine kinase 17a | 9263 |
| STK17B | NM_004226 | serine/threonine kinase 17b | 9262 |
| STK19 | NM_032454 | serine/threonine kinase 19 | 8859 |
| STK24 | NM_003576 | serine/threonine kinase 24 (STE20 homolog, yeast) | 8428 |
| STK25 | NM_006374 | serine/threonine kinase 25 (STE20 homolog, yeast) | 10494 |
| STK3 | NM_006281 | serine/threonine kinase 3 (STE20 homolog, yeast) | 6788 |
| STK32A | NM_145001 | serine/threonine kinase 32A | 202374 |
| STK32B | NM_018401 | serine/threonine kinase 32B | 55351 |
| STK32C | NM_173575 | serine/threonine kinase 32C | 282974 |
| STK33 | NM_030906 | serine/threonine kinase 33 | 65975 |
| STK35 | NM_080836 | serine/threonine kinase 35 | 140901 |
| STK36 | NM_015690 | serine/threonine kinase 36, fused homolog (Drosophila) | 27148 |
| STK38 | NM_007271 | serine/threonine kinase 38 | 11329 |
| STK38L | NM_015000 | serine/threonine kinase 38 like | 23012 |
| STK39 | NM_013233 | serine threonine kinase 39 (STE20/SPS1 homolog, yeast) | 27347 |
| STK4 | NM_006282 | serine/threonine kinase 4 | 6789 |
| STK40 | NM_032017 | serine/threonine kinase 40 | 83931 |
| STYK1 | NM_018423 | serine/threonine/tyrosine kinase 1 | 55359 |
| SYK | NM_003177 | spleen tyrosine kinase | 6850 |
| TAF1 | NM_138923 | TAF1 RNA polymerase II, TATA box binding protein (TBP)-associated factor, 250kDa | 6872 |
| TAF1L | NM_153809 | TAF1-like RNA polymerase II, TATA box binding protein (TBP)-associated factor, 210kDa | 138474 |
| TAOK1 | NM_020791 | TAO kinase 1 | 57551 |
| TAOK2 | NM_016151 | TAO kinase 2 | 9344 |
| TAOK3 | NM_016281 | TAO kinase 3 | 51347 |
| TBK1 | NM_013254 | TANK-binding kinase 1 | 29110 |
| tcag7.1314 | NM_017439 | hypothetical protein LOC54103 | 54103 |
| tcag7.875 | XM_372002 | amyotrophic lateral sclerosis 2 (juvenile) chromosome region, candidate 2 pseudogene | 389599 |
| TEC | NM_003215 | tec protein tyrosine kinase | 7006 |
| TEK | NM_000459 | TEK tyrosine kinase, endothelial (venous malformations, multiple cutaneous and mucosal) | 7010 |
| TESK1 | NM_006285 | testis-specific kinase 1 | 7016 |
| TESK2 | NM_007170 | testis-specific kinase 2 | 10420 |
| TEX14 | NM_031272 | testis expressed 14 | 56155 |
| TGFBR1 | NM_004612 | transforming growth factor, beta receptor I (activin A receptor type II-like kinase, 53kDa) | 7046 |
| TGFBR2 | NM_003242 | transforming growth factor, beta receptor II (70/80kDa) | 7048 |
| TIE1 | NM_005424 | tyrosine kinase with immunoglobulin-like and EGF-like domains 1 | 7075 |
| TK1 | NM_003258 | thymidine kinase 1, soluble | 7083 |
| TK2 | NM_004614 | thymidine kinase 2, mitochondrial | 7084 |
| TLK1 | NM_012290 | tousled-like kinase 1 | 9874 |
| TLK2 | NM_006852 | tousled-like kinase 2 | 11011 |
| TNIK | NM_015028 | TRAF2 and NCK interacting kinase | 23043 |
| TNK1 | NM_003985 | tyrosine kinase, non-receptor, 1 | 8711 |
| TNK2 | NM_001010938 | tyrosine kinase, non-receptor, 2 | 10188 |
| TNNI3K | NM_015978 | TNNI3 interacting kinase | 51086 |
| TPD52L3 | NM_001001874 | tumor protein D52-like 3 | 89882 |
| TPK1 | NM_001042482 | thiamin pyrophosphokinase 1 | 27010 |
| TPR | NM_003292 | translocated promoter region (to activated MET oncogene) | 7175 |
| TRAF3IP3 | NM_025228 | TRAF3 interacting protein 3 | 80342 |
| TRIB1 | NM_025195 | tribbles homolog 1 (Drosophila) | 10221 |
| TRIB2 | NM_021643 | tribbles homolog 2 (Drosophila) | 28951 |
| TRIB3 | NM_021158 | tribbles homolog 3 (Drosophila) | 57761 |
| TRRAP | NM_003496 | transformation/transcription domain-associated protein | 8295 |
| TSKS | NM_021733 | testis-specific kinase substrate | 60385 |
| TSSK1B | NM_032028 | testis-specific serine kinase 1B | 83942 |
| TSSK2 | NM_053006 | testis-specific serine kinase 2 | 23617 |
| TSSK3 | NM_052841 | testis-specific serine kinase 3 | 81629 |
| TSSK4 | NM_174944 | testis-specific serine kinase 4 | 283629 |
| TSSK6 | NM_032037 | testis-specific serine kinase 6 | 83983 |
| TTBK1 | NM_032538 | tau tubulin kinase 1 | 84630 |
| TTBK2 | NM_173500 | tau tubulin kinase 2 | 146057 |
| TTC33 | NM_012382 | tetratricopeptide repeat domain 33 | 23548 |
| TTK | NM_003318 | TTK protein kinase | 7272 |
| TWF1 | NM_002822 | twinfilin, actin-binding protein, homolog 1 (Drosophila) | 5756 |
| TWF2 | NM_007284 | twinfilin, actin-binding protein, homolog 2 (Drosophila) | 11344 |
| TXK | NM_003328 | TXK tyrosine kinase | 7294 |
| TXNDC3 | NM_016616 | thioredoxin domain containing 3 (spermatozoa) | 51314 |
| TXNDC6 | NM_178130 | thioredoxin domain containing 6 | 347736 |
| TYK2 | NM_003331 | tyrosine kinase 2 | 7297 |
| TYRO3 | NM_006293 | TYRO3 protein tyrosine kinase | 7301 |
| UCK1 | NM_031432 | uridine-cytidine kinase 1 | 83549 |
| UCK2 | NM_012474 | uridine-cytidine kinase 2 | 7371 |
| UCKL1 | NM_017859 | uridine-cytidine kinase 1-like 1 | 54963 |
| UHMK1 | NM_175866 | U2AF homology motif (UHM) kinase 1 | 127933 |
| ULK1 | XM_001133335 | unc-51-like kinase 1 (C. elegans) | 8408 |
| ULK2 | NM_014683 | unc-51-like kinase 2 (C. elegans) | 9706 |
| ULK3 | XM_001134013 | unc-51-like kinase 3 (C. elegans) | 25989 |
| ULK4 | XM_929989 | unc-51-like kinase 4 (C. elegans) | 54986 |
| VRK1 | NM_003384 | vaccinia related kinase 1 | 7443 |
| VRK2 | NM_006296 | vaccinia related kinase 2 | 7444 |
| VRK3 | NM_001025778 | vaccinia related kinase 3 | 51231 |
| WEE1 | NM_003390 | WEE1 homolog (S. pombe) | 7465 |
| WNK1 | NM_018979 | WNK lysine deficient protein kinase 1 | 65125 |
| WNK2 | NM_006648 | WNK lysine deficient protein kinase 2 | 65268 |
| WNK3 | NM_001002838 | WNK lysine deficient protein kinase 3 | 65267 |
| WNK4 | NM_032387 | WNK lysine deficient protein kinase 4 | 65266 |
| XYLB | NM_005108 | xylulokinase homolog (H. influenzae) | 9942 |
| YES1 | NM_005433 | v-yes-1 Yamaguchi sarcoma viral oncogene homolog 1 | 7525 |
| YSK4 | NM_001018046 | yeast Sps1/Ste20-related kinase 4 (S. cerevisiae) | 80122 |
| ZAK | NM_133646 | sterile alpha motif and leucine zipper containing kinase AZK | 51776 |
| ZAP70 | NM_001079 | zeta-chain (TCR) associated protein kinase 70kDa | 7535 |
| ZC3HC1 | NM_016478 | zinc finger, C3HC-type containing 1 | 51530 |
| ZMYND8 | NM_012408 | zinc finger, MYND-type containing 8 | 23613 |
